# Traditional Chinese medicine could play an important role in diabetes management: Commentary on “National Chinese medicine guideline for the prevention and treatment of diabetes in primary care (2022)”

**DOI:** 10.1111/1753-0407.13532

**Published:** 2024-04-07

**Authors:** Liyan Jia, Chen Shen, Baoyong Lai, Caoxin Huang, Nengjiang Zhao, Bo Li, Zhihai Zhang, Miaona Cai, Bing Yan, Jianping Liu, Shuyu Yang

**Affiliations:** ^1^ The First Affiliated Hospital of Xiamen University Xiamen China; ^2^ Beijing University of Chinese Medicine Beijing China; ^3^ Extrathoracic Breast Department Beijing University of Chinese Medicine Xiamen Hospital Xiamen China; ^4^ Xiamen Diabetes Institute, Department of Endocrinology and Metabolism The First Affiliated Hospital of Xiamen University Xiamen China; ^5^ Department of Endocrinology and Diabetes The First Affiliated Hospital of Xiamen University Xiamen China; ^6^ Center of Integrated Traditional Chinese and Western Medicine, Medical College, Xiamen University Xiamen China

According to the latest data from International Diabetes Federation, China has the largest number of adults with diabetes, with ~140.9 million people having the disease.^1^ Traditional Chinese medicine (TCM) is one of the earliest complementary and alternative medicines worldwide to explore the prevention and treatment of diabetes.^2^ Nowadays, TCM is being increasingly utilized as adjuvant therapy for the treatment of diabetes. However, there is a lack of TCM clinical guidelines for diabetes in primary health care, which can provide standardized suggestions to physicians. Fortunately, with the support of the State Administration of Traditional Chinese Medicine, a guideline titled *National Chinese medicine guideline for the prevention and treatment of diabetes in primary care* has been developed. The guideline working group systematically reviewed the clinical research evidence related to diabetes and assessed the overall quality of available evidence using the Grading of Recommendations, Assessment, Development, and Evaluations (GRADE) approach. Finally, the guideline was published in Chinese in December 2022.^3^ In order to enhance readers' understanding of this guideline and offer internationally representative insights on the use of TCM for diabetes in primary care, we have conducted a comprehensive commentary from the perspective of the working group.

## 1 INTEGRATING TCM INTO COMPREHENSIVE APPROACHES FOR THE MANAGEMENT OF DIABETES

**FIGURE 1:**
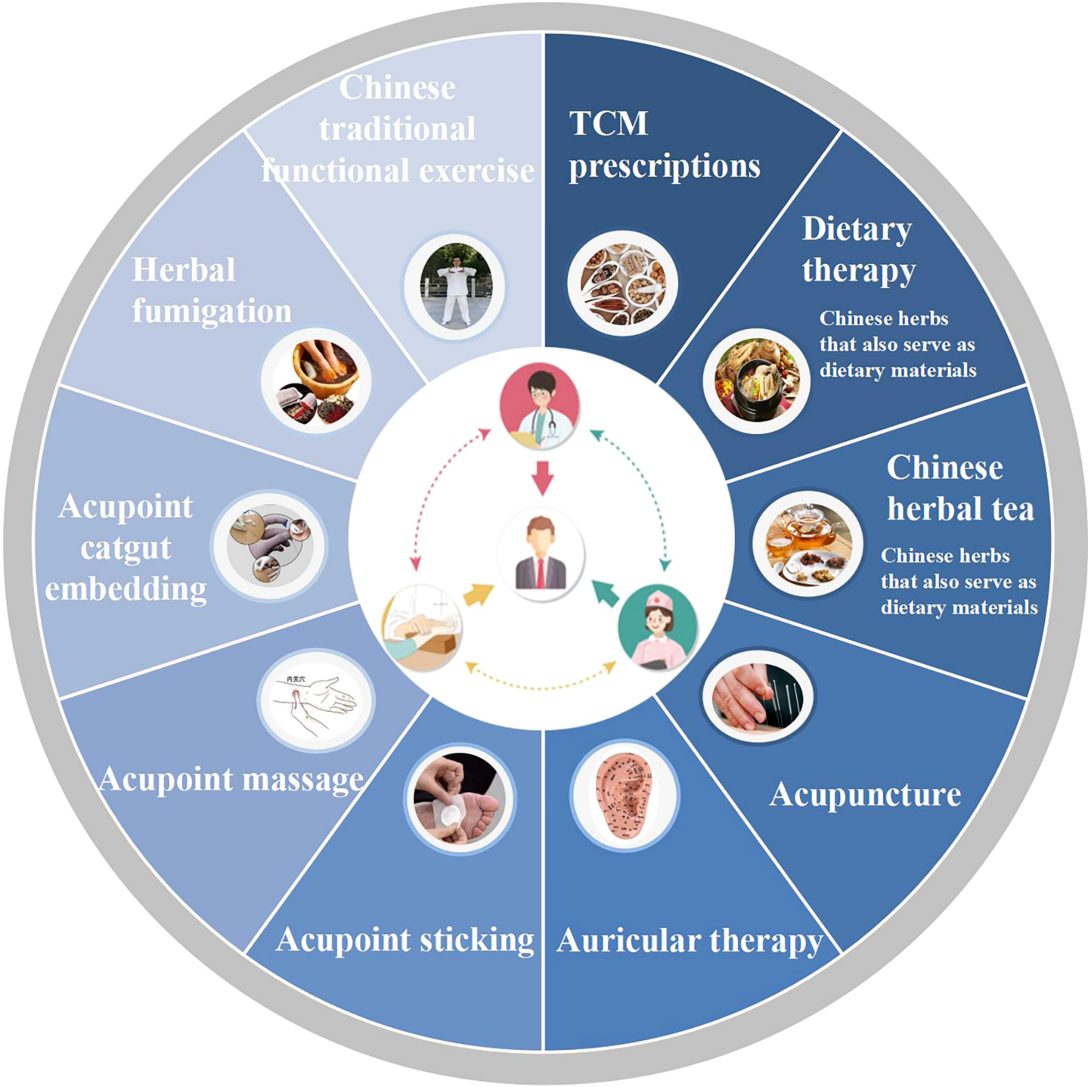
Schematic for recommended Chinese medicine interventions in the guideline. TCM, traditional Chinese medicine.

Diabetes is a complex clinical syndrome characterized by chronic hyperglycemia. In recent years, TCM interventions have been increasingly deployed for the treatment of diabetes. The integration of TCM and Western medicine in treatment has become a significant policy and guideline at a national level in China. Rich clinical research evidence emerging from recent years has shown that TCM therapies are beneficial for the comprehensive prevention and treatment of diabetes, particularly when combined with Western medicine, where it can play a significant role in enhancing effectiveness. Previous studies have reported that TCM has the potential to improve clinical outcomes (such as weight loss, patient's self‐reported symptoms, glucose metabolism) and delay the progression of diabetes.^4^, ^5^ This guideline provides a comprehensive summary of TCM therapies for the prevention and treatment of diabetes, including prevention strategies, nondrug therapies, and external treatment, and so forth. (Figure 1). Throughout this process, we consolidated evidence‐based medical data pertaining to the use of TCM therapies in treating diabetes. The primary medical evidence collected has been compiled and displayed in Appendix S1. Moreover, many Chinese herbs are commonly used as dietary materials in China, which are approved by the National Health Commission (such as Radix Astragali seu Hedysari, Codonopsis pilosula, Radix Rehmanniae Recens, Radix Puerariae Radix Ophiopogonis, and so forth.). Given their beneficial and safe role in managing diabetes, these herbs are suggested in this guideline as part of dietary therapy or as ingredients for Chinese herbal tea. Additionally, representative mechanistic studies have delved into exploring the underlying workings of TCM‐related therapies in treating diabetes. Essential findings from these studies have been compiled and summarized, with the salient points being presented in Appendix S2.

Most important, previous studies have indicated that the majority of patients with diabetes have multiple comorbid conditions, and multidisciplinary care strategies have been found to be highly effective in managing diabetes.^6^ Therefore, this guideline advocates a multidisciplinary management model composed of endocrinologists, TCM practitioners and health management personnel, referred to as the “comanagement of three disciplines” diagnosis‐treatment model, for the management of diabetes.^7^ This management model has been successfully implemented in multiple provinces and cities in China, such as Xiamen, Shenzhen, Anhui, Beijing, Shanghai, Jiangsu, Yunnan, and so forth, receiving positive responses. It carries a valuable significance in integrating TCM into comprehensive prevention and treatment approaches for diabetes.

## 2 FOCUS ON ALLEVIATING PATIENT'S SELF‐REPORTED SYMPTOMS

Most patients with diabetes suffer from both the disease itself and a variety of self‐reported symptoms, such as fatigue, numbness, tingling sensations, constipation, insomnia, nausea, depression, and so forth. Symptom management is one of the key points of TCM in the prevention and treatment of chronic diseases, aiming to optimize the quality of life (QoL) for patients. Previous studies have demonstrated the potential advantages of TCM in alleviating such self‐reported symptoms in patients with diabetes.^8^, ^9^, ^10^

A meta‐analysis, comprising 27 randomized controlled trials (RCTs) and 2490 patients experiencing cool numb pain in limbs, revealed that TCM formulas (Buyang Huanwu Decoction, Danggui Sini Decoction, and Decoction of Five Drugs Including Astragalus and Cinnamon) combined with Western medicine could effectively improve nerve conduction velocity and overall clinical efficacy.^8^ In another RCT involving 60 patients with diabetic diarrhea, a combination of Shenling Baizhu Powder and pirenzepine bromide treatment was shown to be more effective than pirenzepine bromide treatment alone. This combined treatment with TCM significantly improved the patient's gastrointestinal motility, promoted gastric emptying, alleviated clinical symptoms, and lowered the recurrence rate.^9^ Furthermore, to treat constipation in diabetic patients, a prescription consisting of TCM ingredients—rhubarb, orange peel, and magnolia bark, is ground into a powder, mixed with water, and applied to the Shenque acupoint in a paste form that is wrapped in gauze.^10^ Modern pharmacology has confirmed that rhubarb contains substances such as rhubarb tannins and emodin, which can stimulate the intestinal wall, excite smooth muscles, block ion transport channels on the cell membranes of the intestinal wall, increase osmotic pressure within the intestines, and retain water in the intestinal tract. This may be one of the important mechanisms for treating constipation. Research has also suggested the use of auricular acupressure (Wang Buliuxing seeds are applied to acupressure points like liver, gallbladder, heart, subcortex, Shenmen, and sympathetic ear points) for the treatment of insomnia symptoms in patients with diabetes.^11^, ^12^

Moreover, a single‐blind RCT found that diabetes symptom management could improve glycated hemoglobin (HbA1c) levels, glycemic control and QoL and prevent the progression of diabetes.^13^ According to the findings of a nationwide survey of 1150 physicians, the “symptom improvement” is one of the most significant advantages of TCM in treating diabetes. During the meticulous development of the guidelines, expert consensus acknowledged the positive effects of TCM in alleviating 13 self‐reported symptoms related to diabetes. These symptoms include constipation, cold numbness and pain of limbs, sweating, abdominal distention, thirst, fatigue, insomnia, decreased appetite, diarrhea, polyuria, feeling hungry and overeating, pruritus, anxiety, and depression.^14^, ^15^ Consequently, from the perspective of TCM syndrome differentiation, the guideline has carefully compiled a series of comprehensive recommendations for the management of self‐reported symptoms.

## 3 PREVENTION STRATEGIES FOR INDIVIDUALS AT HIGH RISK OF DIABETES AND THOSE WITH PREDIABETES

Before the diagnosis of type 2 diabetes, there is often a long presymptomatic phase. Research has shown that individuals who are obese, suffer from hypertension, or have dyslipidemia are significantly more prone to developing diabetes. It has been observed that these same risk factors are also closely linked to prediabetes. This underscores the importance of identifying and addressing these high‐risk factors related to diabetes as early as possible. Moreover, implementing effective interventions to prevent the progression from the presymptomatic phase or prediabetes to diabetes is crucial, as it can help delay the disease progression.^16^ TCM prevention strategies emphasize the concepts of “prevention before illness” and “stopping the progress of disease,” which has prompted increasing interest in developing TCM strategies to mitigate the high risk of diabetes.^17^ Previous studies have demonstrated the effectiveness of specifically TCM decoction, acupuncture, acupoint catgut embedding, and other external treatments in managing obesity, hypertension, and hyperlipidemia.^18^, ^19^, ^20^ Therefore, the guideline also suggested specific measurements (such as specific TCM prescriptions, auricular therapy, acupoint catgut embedding, acupuncture) as adjuvant therapy to address the high‐risk factors associated with diabetes.

In this guideline, the advantages of TCM prevention strategies for prediabetes were summarized as well. Previous studies, such as a multicenter RCT recruiting patients with impaired glucose tolerance (IGT), have demonstrated that the incorporation of TCM measures into standard healthcare resulted in a substantial reduction in the conversion rate from IGT to diabetes, along with an improvement in insulin resistance.^21^ Additionally, systematic reviews have indicated that traditional Chinese exercises have the potential to delay the progression of diabetes and to improve fasting blood glucose, 2‐hour blood glucose, and HbA1c levels in patients with prediabetes.^22^ Consequently, TCM interventions (including specific prescriptions, acupuncture, acupoint catgut embedding, acupoint massage, traditional Chinese exercises, and so forth.) were suggested as adjuvant therapy for individuals with prediabetes in the guideline.

## 4 UTILIZING TCM MANAGEMENT TECHNIQUES TO PREVENT OR RELIEVE COMPLICATIONS

Diabetes mellitus, a chronic systemic metabolic disease, is commonly accompanied by multiple complications that significantly reduce patients' QoL and increase mortality rates. As we all know, diabetes peripheral neuropathy, diabetes retinopathy and diabetes nephropathy are common chronic complications among patients with diabetes. Despite the existence of numerous mono‐ and combination therapies, the progression of diabetes complications continues to be a global health concern due to the current lack of effective treatments that can reverse it. TCM show promise as a potential complementary approach for managing diabetes complications.^23^ Some classical TCM prescriptions with outstanding curative effect have been in use for hundreds of years and some have been developed into modern medicinal preparations for the treatment of diabetes with evidence of therapeutic effects.^24^ Besides, TCM nondrug therapies and external treatments, which have relatively minor side effects, are also included in the TCM treatment approach for diabetes complications.

Previous meta‐analyses have demonstrated that the Chinese patent medicines (including Compound Danshen Dripping Pills, Compound Xueshuantong Capsule, Qiming granule) as an add‐on therapy for diabetic retinopathy have additional benefits and are generally safe. Chinese patent medicines combined with calcium dobesilate could improve retinal microaneurysm, hemorrhage, macular thickness, visual acuity, fasting blood glucose, and HbA1c compared with calcium dobesilate alone.^25^ Another network meta‐analysis that included 41 RCTs found that the treatment plan of Dihuang pill prescriptions combined with conventional treatment could reduce serum creatinine, 24‐h urinary protein, and fasting blood glucose urine protein excretion rate and improve the total clinical effective rate; the combination of medicine was obviously better than conventional medicine alone.^26^ As for TCM foot baths and acupoint massages, they may promote microcirculation, enhance skin permeability, and increase drug absorption. Therefore, the application of foot baths and acupoint massages holds significant value for patients suffering from cold numbness and pain in their limbs.^27^ Additionally, systematic reviews have suggested the potential benefit of acupuncture in the treatment of diabetic retinopathy.^28^ Similarly, TCM nebulization therapy for eye fumigation and ocular acupoint massage have shown a potential role in improving overall clinical outcomes, visual evoked potentials, visual acuity, and the incidence rate of retinopathy complications.^29^ As a result, TCM measures for related complications (including diabetic peripheral neuropathy, diabetic retinopathy, and diabetic nephropathy) were respectively recommended as adjuvant therapy in this guideline.

## 5 SUMMARIZING EXTERNAL AND NONPHARMACOLOGICAL THERAPIES OF TCM

Currently, TCM external and nonpharmacological therapies also have contributed a lot to the management of diabetes. Previous research has demonstrated that, in addition to TCM decoction, nonpharmacological techniques have shown beneficial effects, practicality, and high demand.^27^, ^28^, ^29^, ^30^, ^31^ For example, acupoint sticking achieves therapeutic effects by stimulating specific acupoints and penetrating the skin, which avoids gastrointestinal irritation and other side effects associated with oral drug administration.^32^ Acupuncture at acupoints is believed to activate peripheral afferent nerve fibers and receptors, leading to the production of anti‐inflammatory, neuroendocrine, and neuroimmune signals, thereby playing a beneficial regulatory role.^33^ Indeed, nonpharmacological therapies can help reduce the medication burden for patients with diabetes. Therefore, nonpharmaceutical therapy is urgently expected as adjuvant therapy to diabetes. Inspirationally, results from a nationwide hierarchical analysis of 1150 physicians revealed a high demand for primary care practitioners to utilize TCM treatment measures, especially nonpharmacological therapies, for diabetes.^15^ Therefore, TCM external treatments (such as foot baths and acupoint sticking) as well as non‐pharmacological therapies (including acupuncture, auricular therapy, acupoint catgut embedding, and acupoint massage) are suggested to varying degrees for the management of diabetes in the guideline. Additionally, regular exercise is considered one of the important ways to prevent and manage diabetes. Therefore, physical exercises related to TCM elements (including Xinshenzhuang exercise, Baduanjin, 24‐Style Simplified Tai Chi, Wuqinxi exercise, Yijinjing, and so forth.^34^, ^35^) are advocated as adjuvant interventions for diabetes in this guideline as well.

In conclusion, *National Chinese medicine guideline for the prevention and treatment of diabetes in primary care* is the first national diabetes guideline for primary care using TCM interventions. This commentary highlights the role of TCM as an adjuvant approach in preventing and treating diabetes, its symptoms, and its related complications. TCM indicated potential benefits in preventing the disease, alleviating related symptoms, managing complications, and delaying disease progression when integrated with modern medical practices. Additionally, more rigorously designed and well‐reported studies are required to confirm the effectiveness and safety of specific TCM measures for diabetes.

## CONFLICT OF INTEREST STATEMENT

All authors declare that they have no conflicts of interest.

## Supporting information


**Appendix S1.** Summary of evidence from research on TCM interventions for prediabetes, type 2 diabetes and its complications.

**Appendix S2.** Representative examples of TCM interventions and it's potential mechanisms.
