# Supplementary material for: Traditional Chinese medicine could play an important role in diabetes management: Commentary on “National Chinese medicine guideline for the prevention and treatment of diabetes in primary care (2022)”
Source: J Diabetes. 2024 Apr 7;16(4):e13532. doi: 10.1111/1753-0407.13532 (PMC10999495; doi:10.1111/1753-0407.13532)
Supplement: Supplementary file 1 — Appendix S1. Summary of evidence from research on TCM interventions for prediabetes, type 2 diabetes and its complications. [file JDB-16-e13532-s002.docx]

Appendix 1. Summary of Evidence from Research on TCM Interventions for Prediabetes, Type 2 Diabetes and Its Complications

| **Study ID** | **Included studies (N)** | **Participants (N)** | **Intervention** | **Control** | **Outcome and main findings (95% confidence interval (CI))** |
| --- | --- | --- | --- | --- | --- |
| **Type 2 diabetes mellitus (T2DM) - Chinese herbal prescription or Chinese patent medicine** | | | | | |
| Zhang Zehua 2022 ^[1]^ | 17 RCTs | 1525 | Dachaihu decoction alone or Dachaihu decoction combined with conventional treatment | Placebo or conventional treatment | Compared with conventional treatment, combined treatment with Dachaihu decoction was significantly better in improving HbA1c (MD = -0.90% [-1.20, -0.60], p < 0.01), FBG (MD = -1.08 mmol/L [-1.28, -0.87], p < 0.01), 2hPG (MD = -1.25 mmol/L [-1.42, -1.09], p < 0.01), TC (MD = -0.50 mmol/L [-0.70, -0.30], p < 0.01), TG (MD = -0.44 mmol/L [-0.61, -0.26], p < 0.01), LDL-C (MD = -0.58 mmol/L [-0.85, -0.31], p < 0.01), HOMA-IR (SMD = -2.04 [-3.09, -0.99], p < 0.01), HOMA-β (SMD = 2.48 [2.20, 2.76], p < 0.01) and BMI (MD = -1.52 kg/m^2^ [-2.55, -0.49], p < 0.01). Based on this meta-analysis, we found that the combination with Dachaihu decoction in the T2DM treatment has more advantages than conventional treatment alone, which can further regulate the glucose and lipid metabolism, reduce insulin resistance, improve islet function and lower BMI. However, more high-quality studies are still needed to provide more reliable evidence for the clinical application of Dachaihu decoction. |
| Ryuk Jin Ah 2017 ^[2]^ | 5 RCTs | 499 | Gegen Qinlian decoction combined with metformin | Metformin | The meta-analysis showed the OR of favorable Gegen Qinlian decoction effect on the marked effectiveness of glycemia (n=499, OR = 2.34 [1.63, 3.37], P < 0.001). The Gegen Qinlian decoction and metformin had a synergistic effect on glycemic control in comparison to metformin alone as a T2DM therapy. More rigorous and larger studies are needed. |
| Hu Zhipeng 2021 ^[3]^ | 14 RCTs | 1586 | Jingui Shenqi Wan alone or JGSQW and hypoglycemic agents | hypoglycemic agents | Pooled results indicated that combination treatment results in a reduction in HbA1c (MD = -0.49% [-0.67, -0.31], FBG (MD = -0.84 [-1.19, -0.49], and 2hBG (MD = -1.38 [-1.60, -1.16]. Sensitivity analyses indicated that these results were robust. Therefore, Compared with hypoglycemic agents alone, combination treatment with Jingui Shenqi Wan enhances the effect on glucose metabolism in patients with T2DM. |
| Peng Sihan 2021 ^[4]^ | 10 RCTs | 871 | Yuquan Pill on the basis of the control group | Western medicine | Analysis showed that the combination of Yuquan Pill and conventional treatment was more effective than conventional treatment alone with regard to the levels of FBG (WMD = -0.83 [-1.01, -0.66], p < 0.001), 2hPG (WMD = -1.40 [-1.49, -1.31], p < 0.001), HbA1c (WMD = -0.87 [-1.26, -0.49], p < 0.001), TC (WMD = -0.50 [-0.61, -0.39], p < 0.001), and overall effective rate (RR = 1.21 [1.12, 1.31], p < 0.001). Hence, evidence suggested that Yuquan Pill might improve glucose and lipid metabolism and inflammation in patients with T2DM. Serious adverse events were not reported. More high-quality RCTs are now needed to verify these findings. |
| Zhou Baochang 2021 ^[5]^ | 38 RCTs | 3757 | Xiaoke decoction alone or combined with hypoglycemic drugs or insulin | Hypoglycemic  drugs or insulin | The Xiaoke decoction groups surpassed the western medicine groups regarding improvement in total efficiency (OR = 3.49 [2.78, 4.39], P < 0.001), and reduction in FPG levels (MD = -1.14 [-1.36, -0.92], P < 0.001), 2hPG (MD = -1.40 [-1.61, -1.19], P < 0.001), HbA1c (MD = -0.77 [-0.95, -0.58], P < 0.001), TCM syndrome score (MD = -4.90 [-7.22, -2.57], P < 0.001). Therefore, adding Xiaoke decoction with western medicine has more advantages for the treatment of T2DM with respect to total efficiency, FPG, 2hPG, HbAlc, and TCM syndrome score. |
| Zheng Yujiao 2021 ^[6]^ | 5 RCTs | 548 | Chinese herbal medicine, Chinese patent medicine, or single Chinese medical herbs, which can be administered in the form of decoctions, granules,or powders | Western medicine or placebo, or no treatment | The results showed there were statistically significant improvements in HbA1c (MD = -0.69 [-0.24, -0.14], p = 0.01, I^2^ = 86%), FBG (MD = -0.87 [-1.26, -0.49], p < 0.001, I^2^ = 75%) and 2hPG (MD = -0.83 [-1.01, -0.65], p < 0.001, I^2^ = 0%). In addition, there were also statistically significant improvements in HOMA-IR (SMD = -0.99 [-1.25, -0.73], p < 0.001, I^2^ = 0%) and HOMA-β (SMD = 0.54 [0.21, 0.87], p = 0.001, I^2^ = 0%). There was a significant change in the relative abundance of bacteria in the genera Bacteroides (SMD = 0.87% [0.58, 1.16]. Hence, TCM had the effect of modulating gut microbiota and improving glucose metabolisms in T2DM patients, further well-conducted studies on TCM interventions targeting the gut microbiota are needed. |
| Hu Jiahui 2020 ^[7]^ | 42 RCTs | 4356 | CPMs (Liuwei Dihuang Wan, Xiaoke Wan, Jinlida Granules, Shenqi Jiangtang Capsules, Tianqi Jiangtang Capsules) combined with Metformin | Metformin | The results of the network meta-analysis showed that, compared with metformin, Liuwei Dihuang Pill (MD = -0.85 [-1.08, -0.62]), Xiao Ke Pill (MD = -0.76 [-1.28, -0.25]), Jinlida (MD = -1.16 [-1.59, -0.73]), Shenqi Jiangtang (MD = -1.14 [-1.43, -0.85]) combined with metformin can effectively reduce HbA1c (P < 0.05), and Jinlida has the best effect; Liuwei Dihuang Pill (MD = -1.02 [-1.37, -0.67]), Xiao Ke Pill (MD = -0.71 [-1.42, -0.01]), Jinlida (MD = -2.10 [-2.89, -1.31]), Shenqi Jiangtang (MD = -1.00 [-1.52, -0.48]), Tianqi Jiangtang (MD = -1.05 [-2.02, -0.09]) combined with metformin can effectively reduce FBG, and Jinlida has the best effect; Liuwei Dihuang Pill combined with metformin can reduce TC (MD = -0.96 [-1.42,-0.50]) and TG (MD = -0.45 [-0.78, -0.12]). Therefore, the 5 types of Chinese patent medicine combined with metformin have varying degrees of improvement on blood sugar and blood lipid indicators for T2DM patients. |
| Ma Kaile 2023 ^[8]^ | 64 RCTs | 6204 | CPMs (Shenqi jiangtang granules, Jinlida granules, Tianmai xiaoke tablets, etc.) combined with conventional western medicine | Conventional western medicine | The results showed that Shenqi jiangtang granule combined with sulfonylurea, Shenqi jiangtang granules combined with metformin and Jinlida granules combined with insulin had significant effects on reductions in FBG, 2hPG and clinical efficacy compared with western medicines alone, which included FBG (MD = -2.17 [-2.50, -1.85]), 2hPG (MD = -1.94 [-2.23, -1.65]), and clinical curative effect (OR = 1.73 [0.59, 2.87]). TCM combined with CWM has a very significant effect on treating T2DM compared with CWM alone. |
| Zhao Xuemin 2022 ^[9]^ | 22 RCTs | 2283 | Jinlida granules with metformin | Metformin | Compared with the control groups, significant changes were found in lowering HbA1c (MD = -0.283 [-0.561, -0.004], P = 0.046) in the jinlida groups. The GRADE-assessed evidence quality for the outcomes was moderate. The adjuvant hypoglycemic effect of Jinlida granules on adult Chinese persons with T2DM was statistically found in lowering HbA1c. |
| Li Ruolan 2020 ^[10]^ | 13 RCTs | 1160 | Shenqi Jiangtang granules on the basis of the control group | Western medicine | Meta-analysis results showed that the test group was better than the control group in Clinical efficiency (n = 818, RR = 1.26 [1.18, 1.34], P < 0.001), FBG (n = 1062, MD = -1.18 [-1.29, -1.06], I^2^ = 0%), PBG (n = 1120, MD = -1.74 [-1.95, -1.53], I^2^ = 27%), HbA1c (n = 1100, MD = -1.13 [-1.24, -1.01], I^2^ = 39%). Moreover, the test group with lower adverse reaction rate than the control group (n = 424, MD = 0.21 [0.08, 0.51], I^2^ = 0%). Therefore, Shenqi Jiangtang granules have a certain clinical effect and low adverse reaction rate for the treatment or adjuvant treatment of T2DM. But a large number of large-sample clinical trials are needed to further verify its specific efficacy and safety. |
| Yuming Gu 2018 ^[11]^ | 7 RCTs | 717 | Tianmai Xiaoke Tablet combined with conventional therapy | Placebo or conventional therapy alone | Tianmai Xiaoke Tablet combined with conventional therapy lowered FBG level (MD = -0.68 [-0.90, -0.45], P < 0.001), 2hPG (MD = -1.33 [-1.86, -0.79], P < 0.001), HbA1c (MD = -0.46 [-0.57, -0.36], P < 0.001), and BMI (MD = -0.77 [-1.12, -0.41], P < 0.001). Tianmai Xiaoke Tablet combined with conventional therapy is beneficial for patients with newly diagnosed T2DM. However, well-designed clinical trials are needed in the future. |
| **Type 2 diabetes mellitus - Non drug therapies** | | | | | |
| Li Shuqing 2021 ^[12]^ | 21 RCTs | 797 | Acupuncture (manual acupuncture,electroacupuncture,acupressure, and transcutaneous acupoint electrical stimulation) plus antidiabetic drugs | Antidiabetic drugs alone | The meta-analytic results showed that the acupuncture group had greater reductions in FBG (MD = -6.46 mg/dL [-11.95, -0.98], HOMA-IR (MD = -1.23 [-2.16, -0.31]. No data on the incidence of diabetic complications were found. All acupuncture-related adverse events reported were mild. Therefore, the current evidence suggests that acupuncture, as a complementary therapy to antidiabetic drugs, has a small but statistically significant effect on decreasing FBG and improving insulin resistance. Acupuncture is generally safe in patients with mild diabetes. More evidence for the long-term effects of acupuncture on T2DM is needed. |
| Wang Ying 2022 ^[13]^ | 13 RCTs | 993 | Acupuncture in addition to the control group | Sham acupuncture, western medicine, or no intervention | Meta-analysis results demonstrated the effective rate of clinical symptoms (RR = 1.19 [1.11, 1.28], P < 0.001), BMI (MD = -2.11 [-2.56, -1.66], P < 0.001), FBG (MD = -1.09 [-1.60, -0.59], P < 0.001), HbA1c (MD = -0.58 [-0.95, -0.20], P = 0.002), TG (MD = -0.29 [-0.46, -0.11], P = 0.001), waist circumference (MD = -5.36 [-8.68, -2.05], P = 0.002), body fat rate (MD = -3.59 [-4.28, -2.90], P < 0.001]. Current evidence suggests that acupuncture has advantages in treating obesity combined with T2DM. However, additional large-sample and high-quality research are required. |
| Zhang Xiaolin 2020 ^[14]^ | 10 RCTs | 769 | Chinese massage combined with metformin hydrochloride tablets | Metformin hydrochloride tablets | Compared with metformin hydrochloride tablets, Chinese massage plus baseline treatment can reduce FBG (WMD = -0.33 [-0.54, -0.13], P = 0.002), 2hPG (WMD = -0.52 [-0.70, -0.34), P < 0.001), HbA1c (WMD = 0.12 [0.04, 0.20], P = 0.003), fasting insulin (WMD = -3.59 [-5.56, -1.42], P < 0.001), TCM syndrome score scale (WMD = -4.55 [-7.58, -1.51], P = 0.003), HOMA-IR (WMD = -1.76 [-2.25, -1.27), P < 0.001), BMI (WMD = -1.28 [-1.65, -0.92], P < 0.001), serum total cholesterol (WMD = -1.01 [-1.14, -0.83], P < 0.001), meanwhile, the effective rate was increased (RR = 1.31 [1.21, 1.42], P < 0.001). Therefore, Chinese massage combined with metformin hydrochloride tablet has a synergistic effect. It can not only be used as an auxiliary treatment of T2DM, but also as an important reference way of reducing drug treatment of T2DM, improving clinical efficacy and reducing adverse reactions. |
| Wang Xinzheng 2022 ^[15]^ | 19 RCTs | 1220 | Tai Chi | Regular exercise | The results show that the Tai Chi exercise group is better than the control group in decreasing FBG (MD = -0.79 [-1.73, -0.68], p < 0.001), HbA1c (MD = -1.10 [-1.78, -0.43], p = 0.001), TG (MD = -0.23 [-0.32, -0.15], p < 0.001) and in increasing HDL-C (MD = 0.15 [0.11, 0.20], p < 0.001). Tai Chi exercise therapy shows significant superiority in improving the FBG, HbA1c, TG, and HDL-C in T2DM patients. It can be used as one of the exercise prescriptions for prevention and treatment of T2DM patient. |
| Qin Jiawei 2021 ^[16]^ | 15 RCTs | 1418 | Tai Chi alone or Tai Chi with standard diabetic care | No intervention, usual care, or other exercises | The aggregated results showed that Tai Chi statistically significantly improved QoL measured by the SF-36 on every domains and BMI (MD = -1.53 [-2.71, -0.36], p < 0.001) compared with control group (no intervention; usual care; other exercises). Therefore, Tai Chi could improve QoL and decrease BMI for patients with T2DM. |
| Lingjun Kong 2022 ^[17]^ | 27 RCTs | 2048 | Baduanjin exercise combined with conventional drugs | Usual care, and therapies without Baduanjin exercises | The aggregated results indicated that Baduanjin exercises showed positive effects in psychological well-being (SMD = 0.96 [0.57, 1.36], p < 0.001), depression (SMD = 1.03 [0.08, 1.97], p = 0.03), anxiety (SMD = 0.88 [0.30, 1.46], p = 0.003), and mental health (SMD = 0.72 [0.42, 1.02], p < 0.001). In glycemic control, Baduanjin exercises showed better improvements in FBG (SMD = 0.53 [0.34, 0.72], p < 0.001), HbA1c (SMD = 0.58 [0.41, 0.75], p < 0.001), and 2hPG (SMD = 0.56 [0.08, 1.03, p = 0.02) compared with usual care. Therefore, Baduanjin exercise is a beneficial therapy for T2DM. |
| Piao Chunli 2020 ^[18]^ | 10 RCTs | 761 | Auricular therapy in addition to the control group | Routine comprehensive treatment | The results of the meta-analysis showed, compared with the control group, auricular therapy can significantly improve the FBG (MD = -0.79 [-0.91, -0.67], P<0.001), 2hPG (MD = -1.35 [-1.73, -0.96], P < 0.001) and overall efficacy (OR = 5.53 [3.36, 9.09], P<0.001). Therefore, auricular therapy may have certain advantages in improving diabetes, but more high-quality studies are needed to make the evidence stronger. |
| **Prediabetes - Non-drug therapies** | | | | | |
| Shi Liwei 2018 ^[19]^ | 11 RCTs | 970 | Acupuncture, or acupuncture combined with other therapies | Other therapies outside acupuncture | The included studies show that acupuncture intervention in the pre-diabetes stage has a good therapeutic effect in improving FBG and 2hPG. Acupuncture may have some efficacy in treating pre-diabetes, but its potential efficacy still needs to be confirmed by high-quality RCTs. |
| Du Xuqin 2019 ^[20]^ | 8 RCTs | 791 | Acupoint catgut embedding in addition to the routine comprehensive treatment | Routine comprehensive treatment | The results of the meta-analysis showed, the experimental group was superior to the control group in improving the clinical effectiveness rate of pre-diabetes patients (RR = 1.31 [1.11, 1.54], P = 0.001), reducing FBG (MD = -0.47 [-0.80, -0.13], P = 0.007), reducing 2hPG (MD = -0.54 [-0.90, -0.18], P = 0.003), and reducing HbA1c (MD = -0.74 [-1.22, -0.27], P = 0.002). Sensitivity analysis showed that the meta-analysis results have good stability. Therefore, acupoint catgut embedding in the treatment of pre-diabetes has a better effect than conventional comprehensive treatment, which can improve the clinical effectiveness rate and improve FBG, 2hPG and HbA1c levels in patients. |
| Yang Qi 2022 ^[21]^ | 10 RCTs | 994 | Auricular therapy combined with lifestyle intervention | Lifestyle intervention | The results showed the auricular acupressure subgroup is superior to the control group in reducing FBG (MD = -0.57 [-0.86, -0.28], P = 0.001], 2hPG (MD = -0.87 [-1.19, -0.58], P < 0.001), and HbA1c (SMD = -0.87 [-1.52, -0.22], P = 0.008]. The auricular acupressure combined with Chinese medicine subgroup is superior to the control group in reducing FBG (MD = -0.54 [-0.72, -0.36], P < 0.001), 2hPG (MD = -1.15 [-1.73, -0.57], P < 0.001), and HbA1c (SMD = -0.78 [-1.05, -0.51], P < 0.001). Sensitivity analysis shows good stability of results. |
| Yu Dong-dong 2020 ^[22]^ | 9 RCTs | 485 | Traditional Chinese exercises (Kungfu, Qi Gong, Tai Chi, Wuqinxi, Baduanjin, Yijinjing) in addition to the control group | Basic treatment, usual care, or no treatment | The results showed that traditional Chinese exercises could reduce FBG, 2hPG and HbA1c in patients with prediabetes. The treatment subgroup showed that an intervention of 6 months and the Baduanjin exercise both yielded better results. Baduanjin exercise: FBG (SMD = -0.83 [-1.13, -0.53], P < 0.001); 2hPG (SMD = -0.75 [-0.94, -0.57], P < 0.001); 2hPG (SMD = -0.62 [-0.91, -0.32], P < 0.001); HbA1c (SMD = -0.46 [-0.83, -0.08], P = 0.02). 6 month treatment: FBG (SMD = -0.73 [-1.20, -0.26], P = 0.002); 2hPG (SMD = -0.91 [-1.39, -0.44], P < 0.001); HbA1c (SMD = -0.77 [-1.24, -0.29], P = 0.002). Hence, traditional Chinese exercises may be of potential therapeutic value for patients with prediabetes, as an adjuvant therapy along with other treatments. |
| Zuo Yufei 2023 ^[23]^ | 11 RCTs | 778 | Tai Chi, Baduanjin | Conventional therapy | The meta-analysis showed that Tai Chi and Baduanjin can effectively reduce FBG (SMD = -1.19 [-1.69, -0.69], P < 0.01, I^2^ = 90%), 2hBG (SMD = -0.62 [-0.88,-0.36], P < 0.01, I^2^ = 53%), and HbA1c (SMD = -1.27 [-2.20, -0.33], P < 0.01, I^2^ = 96%) in the pre-diabetes population. Therefore, Tai Chi and Baduanjin can effectively reduce FBG, 2hBG, and HbA1c levels, which is beneficial for blood glucose control in pre-diabetes individuals. |
| **Diabetes peripheral neuropathy (DPN) - Chinese herbal prescription or Chinese patent medicine** | | | | | |
| Fu Han  2023 ^[24]^ | 27 RCTs | 2490 | Chinese herbal formulae (Buyang Huanwu decoction, Danggui Si Ni decoction, Huang Qi Guizhi Wuwu decoction) combined with Mecobalamin tablets | Mecobalamin tablets | The network Meta-analysis showed that in terms of the overall efficacy in the treatment of DPN with TCM prescription and methycobal tablets, the top three Surface Under the Cumulative Ranking Curve (SUCRA) rankings are methylcobal tablets combined with Buyang Huanwu decoction, methylcobal tablets combined with Danggui Si Ni Tang, and methylcobal tablets combined with Huang Qi Guizhi Wuwu Tang. In terms of the sensory of the peroneal nerve and motor conduction speed, the top two SUCRA rankings are methylcobal tablets combined with Huang Qi Guizhi Wuwu Tang, and methylcobal tablets combined with Danggui Si Ni Tang. The combination of TCM prescription and methycobal tablets can effectively improve the overall treatment efficacy of DPN. |
| Liu Huimin 2022 ^[25]^ | 14 RCTs | 1347 | Huang Qi Guizhi Wuwu decoction combined with acupuncture and conventional Western medicine treatment | Conventional Western medicine treatment | Meta-analysis results showed that the use of Huang Qi Guizhi Wuwu decoction combined with acupuncture treatment of DPN has significant clinical effects. It can improve patients' clinical symptoms and signs, and can improve the patients' median nerve Motor Nerve Conduction Velocity (MNCV), Sensory Nerve Conduction Velocity (SNCV), as well as the peroneal nerve MNCV, SNCV, but further verification is still needed for the above results. |
| **Diabetes peripheral neuropathy - External treatment** | | | | | |
| Fu Qinwei 2020 ^[26]^ | 31 RCTs | 3284 | TCM foot bath combined with acupoint massage therapies on the basis of routine treatment | Conventional therapy, oral TCM, other symptomatic treatment of western medicine | The results showed that TCM foot bath combined with acupoint massage was significantly better compared with the control groups in terms of the total effective rate, SNCV, MNCV, and neuropathic syndrome score. No case of adverse effect was reported. These findings show that TCM foot bath combined with acupoint massage may be safer and more effective for the treatment of DPN. However, further research with RCTs of higher quality is required to prove its efficacy and better evidence for clinical treatment. |
| Lu Chunjian 2022 ^[27]^ | 44 RCTs | 3471 | TCM external treatment (acupuncture, Chinese herbal foot bath, acupoint massage, acupoint injection, moxibustion) combined with conventional Western medicine treatment | Conventional Western medicine treatment | The results of the Bayesian network Meta-analysis show that all 5 common methods of TCM external treatment combined with conventional Western medicine are superior to the use of Western medicine alone in improving the total effective rate, with acupuncture being the most effective (OR = 0.20 [0.11, 0.36]); in improving the MNCV, with Chinese herbal acupoint injection being the most effective (MD = -4.50 [-5.91, -3.11]); in improving the SNCV of the median nerve, with moxibustion being the most effective (MD = -9.03 [-12.59, -5.47]); in improving the peroneal nerve MNCV, with moxibustion being the most effective (MD = -6.34 [-9.31, -3.30]); in improving the peroneal nerve SNCV, with acupuncture being the most effective (MD = -5.70 [-8.03, -3.31]). Therefore, acupuncture combined with conventional Western medicine has shown to be more effective for the treatment of DPN, especially in improving the total effective rate and improving the peroneal nerve SNCV, but further verification from more high-quality studies is still needed. |
| Guo Qiang 2021 ^[28]^ | 14 RCTs | 1214 | TCM external treatment (Chinese herbal foot bath, herbal fumigation, acupuncture) combined with ipragliflozin treatment | Ipragliflozin treatment alone | The results of the Meta-analysis showed that the use of TCM external treatment combined with ipragliflozin can improve the clinical efficacy rate, median nerve conduction speed, and Toronto clinical scoring in the treatment of DPN. However, there is a need for more high-quality research to enhance the clinical significance of the evidence. |
| **Diabetes peripheral neuropathy - Non-drug therapies** | | | | | |
| Jiang Hai lun 2020 ^[29]^ | 18 RCTs | 1200 | Acupuncture alone or acupuncture coupled with chemical drug or vitamin B | Chemical drugs, vitamin B | Acupuncture alone improved clinical efficacy and nerve conduction velocity (P < 0.05). Acupuncture combined with vitamin B improved clinical efficacy and nerve conduction velocity of the three peripheral nerves, peroneal nerve, tibial nerve, and median nerve, and decreased the scores of the Toronto clinical scoring system (P < 0.05). Therefore, acupuncture alone and acupuncture combined with vitamin B are more effective in treating DPN compared to vitamin B. However, more high-quality RCTs on acupuncture combined with vitamin B are required to confirm our results. |
| Nash Jane 2019 ^[30]^ | 10 RCTs | 432 | Acupuncture without restrictions on needle duration, manipulation, location or frequency | Vitamin B, inositol, sham acupuncture, or Japanese acupuncture | Improvements in DPN pain symptoms were reported by all studies. Heterogeneity of outcome measures prevented a meta-analysis. Common acupuncture point selections were *Zusanli* and *Sanyinjiao*. Half of the studies used local point selection. Therefore, acupuncture for DPN appears to improve symptoms. However, the application of acupuncture varies greatly, suitably powered studies using appropriate DPN outcome measures are required. |
| **Diabetic retinopathy (DR) - Chinese herbal prescription or Chinese patent medicine** | | | | | |
| Hu Zhipeng 2021 ^[31]^ | 16 RCTs | 1273 | Qiming granule alone or in combination with the conventional treatment | Conventional treatment | Among 16 included studies, Qiming granule combined with conventional treatment was administered 13.5 g daily for a period ranging from 2 to 6 months. Results showed combination therapy was more effective than conventional treatment alone in central macular thickness (WMD = -29.43 [-39.56, -19.29], p < 0.001), optimum corrected vision (SMD = -0.962 [-1.35, -0.57], p < 0.001) and overall effective rate (RR = 1.25 [1.13, 1.35], p < 0.001). Only three studies reported adverse effects. More high-quality RCTs are needed to confirm the efficacy and safety of Qiming granule in DR. |
| Huang Hui 2021 ^[32]^ | 8 RCTs | 524 | Compound Danshen Dripping Pills combined with western medicine (calcium dobesilate, Qianlieqi injection) | Western medicine or placebo only | Meta-analysis showed that the statistical value of the effective rate of DR treatment in the intervention group and control group was (OR = 5.00 [2.84, 8.83], P < 0.001). The statistical value of visual field gray value comparison was (MD = -0.93 [-0.98, -0.89], P < 0.001). The statistical value of hemangioma volume was (MD = -3.16 [-3.48, -2.84], P < 0.001). The statistical value of hemorrhagic plaque area comparison was (MD = -0.65 [-0.97, -0.32], P < 0.001). The statistical value of visual acuity comparison was (MD = 0.15 [0.10, 0.19], P < 0.001). This study demonstrated that the Compound Danshen Dripping Pills combined with western medicine are effective and safe in the treatment of DR. |
| Wang Shuqian 2023 ^[33]^ | 15 RCTs | 1925 | Compound Danshen Dripping Pills combined with Western medicine treatment | Western medicine treatment | The results of the meta-analysis showed that in terms of the total effectiveness (OR = 3.49 [2.09, 5.86]), vision (OR = 10.49 [3.25, 33.82]), macular thickness (OR = 3.85 [1.41, 10.51]), retinal vascular tumors (SMD = -1.93 [-2.93, -0.93]), hemorrhagic lesions (SMD = -1.13 [-1.89, -0.36]), and TCM symptom score (SMD = 5.88 [2.45, 14.10]), the research group was better than the control group (P < 0.05). Therefore, the results suggest that Compound Danshen Dripping Pills can improve the vision and fundus conditions of patients with DR. |
| Zhang Yue hong 2022 ^[34]^ | 19 RCTs | 1568 | CPMs (Compound Danshen Dripping Pills, compound Xueshuantong, Shuangdan mingmu capsule) in combination with calcium dobesilate | Calcium dobesilate alone | The results suggested that compared with calcium dobesilate alone, CPMs plus calcium dobesilate for DR was superior at reducing the microaneurysm volume (MD = -3.37 [-3.59, -3.14]), microaneurysm counts (MD = -2.29 [-2.97, -1.61]), hemorrhage area (MD = -0.79 [-0.83, -0.75]), and macular thickness (MD = -59.72 [-63.24, -56.20]). Participants in CPMs plus calcium dobesilate group also achieved a better vision. No obvious adverse events occurred. Therefore, CPMs as an add-on therapy for DR have additional benefits and be generally safe. Further studies are needed to provide more conclusive evidence. |
| **Diabetic retinopathy - Non-drug therapies** | | | | | |
| Lin Ang 2020 ^[35]^ | 6 RCTs | 502 | Acupuncture (manual acupuncture, electroacupuncture, laser acupuncture, and articularinjection) alone or in combination with standard medication | Standard medication, standard care, or blank control | Four studies reported the beneficial effects of acupuncture with standard medication or acupuncture alone compared with standard medication on the effective rate. Three studies showed that acupuncture combined with standard medications significantly improved visual acuity compared to standard medication alone. None of the studies reported on adverse events. The results suggest the potential benefit of acupuncture in treating DR. Acupuncture in the form of combined therapy with standard medication or acupuncture alone may be more effective in the treatment of DR than standard medication alone. Further rigorous clinical trials are needed to confirm these findings. |
| Xu Jiayu 2021 ^[36]^ | 15 RCTs | 1145 | Acupuncture treatment, or acupuncture combined with TCM treatment or Western medicine treatment | Western medicine treatment or TCM treatment (excluding acupuncture) | The results of the meta-analysis showed that acupuncture treatment for DR can improve TCM symptom score (MD = -7.04 [-10.87, -3.21], P < 0.001), visual acuity (MD = 0.15 [0.09, 0.20], P< 0.001), and the overall effective rate (OR = 2.91 [2.13, 3.98], P < 0.001), which are all better than the control group. Based on the available data, it is proven that the clinical effect of acupuncture treatment for diabetic retinopathy is better than other treatments, especially in terms of improving visual acuity, TCM symptom score, and the overall effectiveness rate. |
| Fan Yi 2023 ^[37]^ | 1 RCT | 120 | Eye acupoint massage, Chinese herbal atomized eye fumigation, and a combination of Chinese herbal atomized eye fumigation with eye acupoint massage, all applied on the basis of the control group treatments | Conventional treatment and usual care | After intervention, the combined group had the best overall effectiveness, vision evoked potential improvement, and vision enhancement. Meanwhile, the occurrence rate of fundus complications in the combined group was the lowest (P < 0.05). Therefore, the results suggest that Chinese herbal atomized eye fumigation combined with eye acupoint massage can effectively improve the clinical symptoms of patients with non-proliferative DR and improve the patients' vision. |
| **Diabetic nephropathy (DN) - Chinese herbal prescription or Chinese patent medicine** | | | | | |
| Liu Wanlei 2023 ^[38]^ | 13 RCTs | 1335 | Modified Shenqi Dihuang Decoction combined with Western medicine intervention | Western medicine intervention | The efficacy of the experimental group was better than the control group (RR = 1.23 [1.16, 1.30], P < 0.01); TCM syndrome score (SMD = -2.43 [-4.07, -0.80], P < 0.01); 24 h urine protein quantification (SMD = -2.28 [-4.21, -0.38], P = 0.02); Blood creatinine (MD = -7.84 [-10.41, -5.27], P < 0.01); Blood urea nitrogen (MD = -0.46 [-0.61, -0.31, P < 0.01); FBG (MD = -0.69 [-1.04, -0.35], P < 0.01); HbA1c (MD = -0.58 [-0.79, -0.37], P < 0.01. Therefore, the combination of Shen Qi Di Huang decoction and western medicine treatment for DN is more effective than using western medicine alone, which can reduce urinary protein, inhibit inflammatory factors, and delay the progression of kidney function damage. |
| Lin Minghao 2023 ^[39]^ | 41 RCTs | 3562 | Jingui Shenqi pills, Jisheng Shenqi pills, Zhibai Dihuang pills, Liuwei Dihuang Pills combined with western medicine | Western medicine | The results showed that the treatment plan of conventional Western medicine combined with CPM could reduce serum creatinine, 24-hour urinary protein, fasting blood glucose urine protein excretion rate and improve the total clinical effective rate. The combination of medicine was obviously better than conventional Western medicine alone. |
| Huang Linglong 2020 ^[40]^ | 15 RCTs | 1216 | Ji Sheng Shen Qi Wan | Conventional treatment | The results of the meta-analysis showed that the effective rate of the observation group was significantly higher than that of the control group (RR = 1.32 [1.23, 1.42], P < 0.01), 24h urinary protein quantification was significantly lower than the control group (MD = -0.16 [-0.21, -0.11], P < 0.01), 2h postprandial blood glucose level was significantly better than the control group (MD = -1.29 [-1.62, -0.96], P < 0.01), and glycosylated hemoglobin content was significantly lower than the control group (MD = -1.32 [-2.08, -0.57], P < 0.01), with all differences being statistically significant. Therefore, Jisheng Shenqi Pills have a good clinical treatment effect for DR. Further rigorously designed high-quality RCTs are still needed. |
| Ye Chun 2021 ^[41]^ | 13RCTs | 1332 | Yiqi Huoxue prescription on the basis of conventional treatment | Conventional treatment | After treatment, compared to the control group, the experimental group exhibited lower urine microalbumin excretion rate (UAER) (MD = -33.94 [-42.60, -25.28], P < 0.001), serum creatinine (SCr) (MD = -7.43 [-11.50, -3.36], P < 0.001), HbA1c (MD = -0.38 [-0.68, -0.08], P = 0.01), TG (MD = -0.44 [-0.76, -0.13], P = 0.006), and TC (MD = -0.37 [-0.57, -0.18], P < 0.001). Furthermore, the experimental group also showed higher effectiveness rate (OR = 3.81 [2.71, 5.35], P < 0.001) after treatment. The included literature had low bias risk. Yiqi Huoxue prescription on the basis of conventional Western medicine can significantly improve the renal function and reduce the levels of blood glucose and blood lipids of patients with diabetic nephropathy. |
| Zhang Xinxia 2021 ^[42]^ | 20 RCTs | 3566 | Integrated traditional Chinese and western medicine, or TCM treatment alone | Conventional western medicine | Meta-analysis results showed that the clinical treatment efficiency of the experimental group was dramatically higher than the control group (MD = 6.22 [3.77, 10.25], P < 0.001). Moreover, the serum creatinine (Scr), blood urea nitrogen (BUN), urine protein excretion rate (UAER), 24 h postoperative urine protein quantification, and tumor necrosis factor-alpha (TNF-α) of patients after TCM intervention were all remarkably inferior to those of the control group as seen in the following results: Scr (MD = -8.69 [-9.92, -7.47], P < 0.001); BUN (MD = -1.74 [- 2.48, -1.00], P < 0.001); UAER (MD = -26.16 [-46.89, -5.44], P = 0.01); 24 h postoperative urine protein quantification (MD = -0.54 [-0.68, -0.4], P < 0.001); TNF-α (MD = -5.3 [-9.15, -1.46], P = 0.007); and high sensitivity C-reactive protein (MD = -1.34 [-1.9, -0.78], P < 0.001). TCM intervention is effective in treating the clinical symptoms of patients with DN. |
| **Diabetic nephropathy - Non-drug therapies** | | | | | |
| Wang Anna 2019 ^[43]^ | 11 RCTs | 1018 | Acupuncture combined with conventional Western medicine treatment | Western medicine treatment | Meta-analysis results showed that, comparing the experimental group with the control group, the difference of total effective rate is statistically significant (OR = 5.46 [3.79, 7.86], P < 0.001). Therefore, the combination of acupuncture and western medicine treatment may be an effective therapeutic strategy for DN, superior to conventional western medicine treatment alone, but still requires more high-quality RCTs to confirm these findings. |

*Note*. TCM, traditional Chinese medicine; T2DM, type 2 diabetes mellitus; RCTs, randomized controlled trials; OR, odds ratio; RR, relative risk; MD, mean difference; SMD, standardized mean difference; WMD, weighted mean difference; HbA1c, glycated hemoglobin; FBG, fasting blood glucose; 2hPG, 2-h postprandial blood glucose; TC, total cholesterol; TG, triglyceride; LDL-C, low-density lipoprotein cholesterol; HDL-C, high-density lipoprotein cholesterol; HOMA-IR, homeostasis model assessment of insulin resistance; HOMA-β, homeostasis model assessment of β-cell function; BMI, body mass index; QoL, quality of life; MNCV, median nerve Motor Nerve Conduction Velocity; SNCV, Sensory Nerve Conduction Velocity; CPMs, Chinese patent medicines; DPN, diabetes peripheral neuropathy; The interval estimation of all the data is 95% confidence interval (CI).

REFERENCES

[1]Zhang Z, Leng Y, Fu X, et al. The efficacy and safety of dachaihu decoction in the treatment of type 2 diabetes mellitus: A systematic review and meta-analysis. *Front Pharmacol*. 2022;13:918681.

[2]Ryuk JA, Lixia M, Cao S, et al. Efficacy and safety of Gegen Qinlian decoction for normalizing hyperglycemia in diabetic patients: A systematic review and meta-analysis of randomized clinical trials. *Complement Ther Med*. 2017;33:6-13.

[3]Hu Z, Liu X, Yang M. Evidence and potential mechanisms of Jin-Gui Shen-Qi Wan as a treatment for type 2 diabetes mellitus: A systematic review and meta-analysis. *Front Pharmacol*. 2021;12:699932.

[4]Peng S, Xie Z, Zhang X, et al. Efficacy and safety of the Chinese patent medicine Yuquan Pill on type 2 diabetes mellitus patients: A systematic review and meta-analysis. *Evid Based Complement Alternat Med*. 2021;2021:2562590.

[5]Zhou B, Zhang G, Guo W, et al. Xiaoke decoction in treatment of type II diabetes: A meta-analysis. Chin Herb Med. 2021;14(1):130-141. [6]Zheng Y, Ding Q, Wei Y, et al. Effect of traditional Chinese medicine on gut microbiota in adults with type 2 diabetes: A systematic review and meta-analysis. *Phytomedicine*. 2021;88:153455.

[7]Hu JH, Qian HN, Bai XF, et al. Network meta-analysis of the effectiveness of different Chinese patent medicines combined with metformin in the treatment of type 2 diabetes. *Journal of Traditional Chinese Medicine*. 2020;61(24):2163-2173.

[8]Ma K, Zhou L, Zhang Y, et al. Efficacy and safety of traditional Chinese medicines combined with conventional Western medicines in the treatment of type 2 diabetes mellitus: a network meta-analysis of randomized controlled trials. *Front Endocrinol (Lausanne)*. 2023;14: 1134297.

[9]Zhao X, Liu L, Liu J. Treatment of type 2 diabetes mellitus using the traditional Chinese medicine Jinlida as an add-on medication: A systematic review and meta-analysis of randomized controlled trials. *Front Endocrinol (Lausanne)*. 2022;13:1018450.

[10]Li RL, Dong TW, Wei JG, et al. Meta-Analysis of the Therapeutic Effect of Shenqi Jiangtang Granule on Type 2 Diabetes Mellitus. *Evid Based Complement Alternat Med*. 2020;2020:5754823.

[11]Gu Y, Xu X, Wang Z, et al. Chromium-containing traditional Chinese medicine, Tianmai Xiaoke Tablet, for newly diagnosed type 2 diabetes mellitus: A meta-analysis and systematic review of randomized clinical trials. *Evid Based Complement Alternat Med*. 2018; 2018:3708637.

[12]Li SQ, Chen JR, Liu ML, et al. Effect and safety of acupuncture for type 2 diabetes mellitus: A systematic review and meta-analysis of 21 randomised controlled trials. *Chin J Integr Med*. 2022;28(5):463-471.

[13]Wang Y, Xu GN, Wan RH, et al. Acupuncture in treating obesity combined with type 2 diabetes mellitus: A systematic review and meta-analysis of randomized controlled clinical trials. *Complement Ther Clin Pract*. 2022;49:101658.

[14]Zhang X, Cao D, Yan M, et al. The feasibility of Chinese massage as an auxiliary way of replacing or reducing drugs in the clinical treatment of adult type 2 diabetes: A systematic review and meta-analysis. *Medicine (Baltimore)*. 2020;99(34):e21894.

[15]Xinzheng W, Fanyuan J, Xiaodong W. The effects of Tai Chi on glucose and lipid metabolism in patients with diabetes mellitus: A meta-analysis. *Complement Ther Med*. 2022;71:102871.

[16]Qin J, Chen Y, Guo S, et al. Effect of Tai Chi on quality of life, body mass index, and waist-hip ratio in patients with type 2 diabetes mellitus: A systematic review and meta-analysis. *Front Endocrinol (Lausanne)*. 2021;11:543627.

[17]Kong L, Ren J, Fang S, et al. Effects of traditional Chinese mind-body exercise-Baduanjin for type 2 diabetes on psychological well-being: A systematic review and meta-analysis. *Front Public Health*. 2022;10:923411.

[18]Piao CL, Bi CR, Jin D, et al. Systematic review and meta-analysis of auricular acupuncture therapy for type 2 diabetes. *Chinese Journal of Gerontology*. 2020;40(2):276-280.

[19]Shi LW, Ni Q, Li XW, et al. Systematic review of interventional effect of acupuncture and moxibustion on impaired glucose regulation. *Shandong J Tradit Chin Med*. 2018;37(4):282-288.

[20]Du XQ, Xie CG, Shi LP, et al. Meta-analysis of acupoint embedding for pre-diabetes. *Acta Chinese medicine*. 2019;34(5):1121-1125.

[21]Yang Q, Jing L, Wang ZW, et al. Meta-analysis ofauricular plaster therapy combined with traditional Chinese medicine for pre-diabetes. *J Hunan Normal Univ(Med Sci)*. 2022;19(6):113-118.

[22]Yu DD, You LZ, Huang WQ, et al. Effects of traditional Chinese exercises on blood glucose and hemoglobin A1c levels in patients with prediabetes: A systematic review and meta-analysis. *J Integr Med*. 2020;18(4):292-302.

[23]Zuo YF. Effects of Tai Chi and Baduanjin on blood glucose control in prediabetes: A systematic review and meta-analysis. *Zhejiang Sport Science*. 2023;45(2):106-112.

[24]Fu H, Zhu HL, Han QN, et al. Network meta-analysis on the efficacy of traditional Chinese medicine combined with mecobalamin tablets for the treatment of diabetic peripheral neuropathy. *Hunan Journal of Traditional Chinese Medicine*. 2023;39(9):139-146.

[25]Liu HM, Song CQ, Liu ZH, et al. A meta-analysis of the effcacy of the Huangqi Guizhi Wuwu decoction plus acupuncture on diabetic peripheral neuropathy. *Clinical Journal of Chinese Medicine*. 2022;14(29):144-148.

[26]Fu Q, Yang H, Zhang L, et al. Traditional Chinese medicine foot bath combined with acupoint massage for the treatment of diabetic peripheral neuropathy: A systematic review and meta-analysis of 31 RCTs. *Diabetes Metab Res Rev*. 2020;36(2):e3218.

[27]Lu CJ, Liu W, Lin SX, et al. Efficacy and safety of the five commonly used external therapies of TCM combined with conventional Western medicine in treating diabetic peripheral neuropathy: a Bayesian network meta-analysis. *Chinese General Practice*. 2022;25(33):4106-4116.

[28]Guo Q, Zhao H, Yan X, et al. Meta-analysis of clinical efficacy and safety of external therapy of traditional Chinese medicine combined with epalrestat in the treatment of patients with diabetic peripheral neuropathy-A systematic review. *World Journal of Integrated Traditional and Western Medicine*. 2021;16(1):17-22.

[29]Jiang HL, Jia P, Fan YH, et al. Manual acupuncture or combination with Vitamin B to treat diabetic peripheral neuropathy: A systematic review and meta-analysis of randomized controlled trials. *Biomed Res Int*. 2020;2020:4809125.

[30]Nash J, Armour M, Penkala S. Acupuncture for the treatment of lower limb diabetic peripheral neuropathy: a systematic review. *Acupunct Med*. 2019;37(1):3-15.

[31]Hu Z, Xie C, Yang M, Fu X, et al. Add-on effect of Qiming granule, a Chinese patent medicine, in treating diabetic macular edema: A systematic review and meta-analysis. *Phytother Res*. 2021;35(2):587-602.

[32]Huang H, Li Y, Huang Q, et al. Efficacy of Compound Danshen Dripping Pills combined with western medicine in the treatment of diabetic retinopathy: a systematic review and meta-analysis of randomized controlled trials. *Ann Palliat Med*. 2021;10(10):10954-10962.

[33]Wang SQ, Xin K, Feng ZH. Meta-analysis of Compound Danshen Dripping Pills in the treatment of diabetic retinopathy. *Chinese medicine modern distance education China*. 2023;21(13):49-52.

[34]Zhang Y, An X, Duan L, et al. Effect of Chinese patent medicines on ocular fundus signs and vision in calcium dobesilate-treated persons with non-proliferative diabetic retinopathy: A systematic review and meta-analysis. *Front Endocrinol (Lausanne)*. 2022;13:799337.

[35]Ang L, Song E, Jun JH, et al. Acupuncture for treating diabetic retinopathy: A systematic review and meta-analysis of randomized controlled trials. *Complement Ther Med*. 2020;52:102490.

[36]Xu Jiayu, Yang Xirui, Liu Qingjiao, et al.Meta-analysis of clinical efficacy and safety of acupuncture for diabetic retinopathy. *Journal of Traditional Chinese Ophthalmology*. 2021;31(4):297-302.

[37]Fan Y, Zhang CL, Chen L, et al. Clinical Study on the intervention of traditional Chinese medicine atomization eye fumigation combined with eye acupoint massage for non-proliferative diabetic retinopathy. *Journal of Guizhou University of Traditional Chinese Medicine*. 2023;45(2):41-45,80.

[38]Liu WL. Systematic review of the treatment of Qi-Yin deficiency type diabetic nephropathy with Shenqi Dihuang Decoction. *TCM Res*. 2023;36(3):73-81.

[39]Lin M, Zhang H, Liu S, et al. Efficacy of "Dihuang pill prescriptions" combined with conventional treatment for diabetic kidney disease: A network meta-analysis and systematic review. *Medicine (Baltimore)*. 2023;102(39):e35290.

[40]Huang LL, Wang GD, Zhang JP, et al. Systematic review of clinical efficacy of Jisheng Shenqi Pills for diabetic nephropathy. *Evaluation and∞alysis of drug-use in hospitals of China*. 2020;20(5):586-592.

[41]Ye C, Gu L, Feng Y, et al. Therapeutic effects of Yiqi Huoxue prescription on diabetic nephropathy: a meta-analysis and systematic review. *Ann Palliat Med*. 2021;10(6):6617-6629.

[42]Zhang X, Wu M, Zhou J, et al. Meta-analysis-based systematic review of effect of traditional Chinese medicine intervention in treatment of diabetic nephropathy on thyroid function. *Ann Palliat Med*. 2021;10(6):6736-6752.

[43]Wang AN, Gao H, Yang YF, et al. A systematic review of acupuncture on diabetic kidney disease. *Guiding Journal of Traditional Chinese Medicine and Pharmacology*. 2019;25(9):81-86.
