# Supplementary material for: Traditional Chinese medicine could play an important role in diabetes management: Commentary on “National Chinese medicine guideline for the prevention and treatment of diabetes in primary care (2022)”
Source: J Diabetes. 2024 Apr 7;16(4):e13532. doi: 10.1111/1753-0407.13532 (PMC10999495; doi:10.1111/1753-0407.13532)
Supplement: Supplementary file 2 — Appendix S2. Representative examples of TCM interventions and it's potential mechanisms. [file JDB-16-e13532-s001.docx]

Appendix 2. Representative Examples of TCM Interventions and It’s Potential Mechanisms

| Formula/Treatment | Herbs/Methods | Beneficial Effects | Potential Mechanisms |
| --- | --- | --- | --- |
| Gegen Qinlian decoction ^[1-4]^ | *Puerariae lobataeradix, Scutellariae radix, Coptidis rhizoma, Glycyrrhizae radix* | Reducing oxidative stress and insulin resistance (IR), anti-inflammation, regulation of intestinal flora | Up-regulation of antioxidant enzymes (SOD, catalase, glutathione peptide peroxidase), and decrease in malondialdehyde (MDA) levels; inhibition of LPS/TNF-±/L-6 pathway; up-regulation of *Bacteroides vulgatus* strain and *Lactobacillus johnsonii* |
| Baihu decoction ^[5-9]^ | *Gypsum Fibrosum, Anemarrhenae Rhizoma, Glycyrrhizae Radix et Rhizoma Praeparata cum Melle, and non-glutinous rice* | Regulating blood glucose and lipid metabolism, reducing islet cell load, alleviating insulin resistance, and promoting insulin sensitivity | Enhance insulin sensitivity, lower blood glucose and lipid levels, improve glucose tolerance, reduce the level of serum inflammatory factors, and regulate liver lipid metabolism by regulating insulin receptor substrate 1 (IRS-1)/PI3K/Akt signaling pathways |
| Banxia Xiexin decoction ^[10-13]^ | *Pinelliae Rhizoma, Zingiberis Rhizoma, Scutellariae Radix, Coptidis Rhizoma, Ginseng Radix et Rhizoma, Jujubae Fructus, and Glycyrrhizae Radix et Rhizoma* | Regulates the liver and spleen function, blood glucose and lipid metabolism, gut microbiota, and neurotransmitters | Improve the proliferation and differentiation of islet β cells, prevent their functional failure, alleviate IR, regulate blood. It can also enhance insulin sensitivity by regulating various cytokines such as TNF-α and interleukin-6, which are secreted by adipose tissue and cellular factors, thereby alleviating the inflammatory reaction and indirectly improving IR |
| Erchen decoction ^[14-17]^ | *Pinelliae Rhizoma, Pericarpium Citri Reticulatae, Poria, Glycyrrhizae Radix et Rhizoma Praeparata cum Melle, Zingiberis Rhizoma Recens, and Mume Fructus* | Regulates Qi, resolves phlegm, reduce body weight, regulate blood glucose and lipid levels, and produces an antioxidation effect, improve glucose tolerance, improve insulin sensitivity | Regulating the SIRT1/ mitochondrial uncoupling protein 2 signaling pathway. Modify insulin signal transduction. Attenuate insulin resistance by acting on the IRS-1/PI3K/ Akt signaling pathways in hepatocytes |
| Yuquan pill ^[18-20]^ | *Pueraria Lobata Radix, Ophiopogonis Radix, Rehmanniae Radix, Schisandrae Chinensis fructus, Trichosanthis Radix, and Glycyrrhizae Radix et Rhizoma* | Relieves thirst and restlessness, invigorates Qi and regulates the stomach, lowers blood glucose, reduces the cellular level of pro-inflammatory factors, protects endothelial cells, and scavenges free radicals | Ophiopogonis Radix polysaccharides have indicated a protective effect against islet β-cell damage caused by alloxan. Glycyrrhizic acid and licorice flavonoids can inhibit α-glucosidase, delay the speed of intestinal absorption of glucose, and produce hypoglycemic effects. The hypoglycemic effect of Rehmannia polysaccharide in Radix Rehmanniae can have various mechanisms including glucose-dependent insulinotropic peptide and GLP-1 which promotes insulin secretion. Glucose-dependent insulinotropic peptide improves insulin sensitivity, while GLP-1 can inhibit glucagon secretion and promote insulin secretion. Trichosanthes kirilowii lectin in Trichosanthis Radix has anti-lipolysis and other insulin-like effects, and fructus Schisandra polysaccharides can regulate the expression of glucose transporter 4 in the cell membrane by upregulating the levels of Akt, PI3K, and IRS1, which then play an anti-IR role. |
| Danggui Liuhuang decoction ^[21, 22]^ | *Angelicae sinensis Radix, Rehmanniae Radix, Rehmanniae Radix Praeparata, Scutellariae Radix, Coptidis Rhizoma, Phellodendri Chinensis Cortex, and Astragali Radix* | Clearing deficiency heat, nourishing Yin and purging fire, resisting liver fibrosis, and inhibiting islet cell apoptosis and immunosuppression | The substances contained in Danggui Liuhuang decoction, such as ferulic acid, Catalpol, Baicalin, Berberine, and Astragaloside IV, can promote glucose uptake in HepG2 cells, inhibit proliferation of T lymphocytes, promote the differentiation of regulatory T cells in vivo, inhibit the interactions between dendritic cells and T lymphocytes, enhance the expression of α1-antitrypsin-1, B-cell lymphoma gene-2 and cyclin D 1, inhibit the expression of Bcl-2 related X protein, and increase the expression of programmed death ligand-1 in DCS, which will delay the incidence and development of diabetes. |
| Yu’nv decoction ^[23-29]^ | *Gypsum Fibrosum, Anemarrhenae Rhizoma, Rehmanniae Radix Praeparata, Ophiopogonis Radix, and Achyranthis Bidentatae Radix* | Clearing stomach heat, nourishing kidney Yin, lowering blood glucose, anti-inflammation, and anti-ventricular remodeling, improve symptoms, have antipyretic and antiinflammatory effects | Both diphenylpyrones mangiferin and neomangiferin in Anemarrhenae Rhizoma lower blood glucose levels. The flavonoid chemical constituents in Rehmanniae Radix have anti-inflammatory and antibacterial effects, and some glycosides can effectively lower blood glucose and improve blood lipid levels. Berberine in Radix Achyranthis Videntatae can improve glucose uptake and utilization, and reduce fat production, glycogen decomposition, and gluconeogenesis. Triterpenoids and ecdysterone can prevent the increase in drug-induced blood glucose levels, reduce blood lipid levels, regulate immunit. |
| Liuwei Dihuang decoction ^[30-32]^ | *Rehmanniae Radix Praeparata, Corni Fructus, Dioscoreae Rhizoma, Moutan Cortex, Alismatis Rhizoma, and Poria* | Regulating immune function, and providing anti-tumor and antiaging effects, prevents and treats abnormal glucose metabolism, reduce the blood glucose index, cholesterol, triacylglycerol, and low-density lipoprotein content | Through upregulating the expression of phosphodiesterase 3B factor in adipose tissue, promote Akt-mediated phosphorylation and activation, reduce the level of cAMP in cells, reduce the activity of protein kinase A, and ultimately reduce the hydrolysis of stored TG and the release of free fatty acids from adipocytes, to alleviate IR. Its water-extract and alcohol-soluble parts can promote the recovery of insulin signaling in adipose tissue and intervene in IR by upregulating the expression of the crucial gene insulin receptor substrate 2, and PI3K and Akt in PI3K/Akt signaling pathways in the adipose tissue, thereby improving diabetes. |
| Jinlida granule ^[33-38]^ | *Radix Ginseng, Rhizoma Polygonati, Rhizoma Atractylodis, Radix Sophorae Flavescentis, Radix Ophiopogonis, Radix Rehmanniae, Radix Polygoni Multiflori, Fructus Corni, Poria, Herba Eupatorii, Rhizoma Coptidis, Rhizoma Anemarrhenae, Herba Epimedi, Radix Salviae Miltiorrhizae, Radix Puerariae, Semen Litchi, Cortex Lycii* | Regulating glucose and lipid metabolism of skeletal muscle, regulating insulin signaling pathway, enhancing mitochondrial function and reducing lipid deposition of skeletal muscle, reducing oxidative stress of skeletal muscle and liver | Up-regulation of the expression of INSR and IRS-2 of skeletal muscle; down-regulation of SREBP-1c and DGAT-1 mRNA and protein levels; INSR-IRS-2-PI3K-AKT/PKB-GLUT4 axis; GH-IGF-1 axis, GH decreased and IGF-1 increased; upregulation of mRNA and protein levels of PCC-± and CPT1; up-regulation of mRNA and protein levels of SIRT3, activating SOD and GSH, inhibiting ROS and MDA; inhibiting JNK and p38MAPK pathways; improving HPT axis dysfunction in DM; inhibiting NF-º B pathway; enhancing the autophagy in NIT-1 pancreatic beta cells mediated by AMPK activation |
| Xiaoke Pill ^[39-41]^ | *Radix Puerariae, Radix Rehmanniae, Radix Astragali, Radix Trichosanthis, Stylus Zeae Maydis, Fructus Schisandrae Sphenantherae, and Rhizoma Dioscoreae* and glibenclamide | Inhibiting absorption of glucose, regulating glucose and lipid metabolism of adipocytes, promoting endothelial repair, maintaining vascular stability | Inhibition of ±-glucosidase; promoting the proliferation and differentiation of adipocytes, promoting the consumption of glucose, reducing the generation of fluorescence fundus angiography; increasing the number of endothelial progenitor cells, activating adhesion, migration |
| Compound Danshen Dripping Pills ^[42-44]^ | *Radix Salviae Miltiorrhizae, Radix Notoginseng,Borneolum Syntheticum* | Reducing oxidative stress of beta cells, reducing islet fibrosis; reducing oxidative stress of kidney, reducing oxidative stress and regulating hemodynamics | Activating SOD and GSH, inhibiting MDA, down-regulating expression of ±-SMA of PSC; inhibiting HIF-1±/VEGF pathways; decreasing PAI-1, increasing t-PA, activating SOD and GSH, inhibiting ROS and MDA |
| *Radix et Rhizoma Rhei* ^[45]^ | Emodin | Alleviating insulin resistance | Up-regulation of PPAR-± and PPAR-³ , activating GLUT-2 and GLUT-4 |
| *Radix Rehmanniae Recens* ^[46]^ | Rehmannia glutinosa polysaccharide | Regulating energy metabolism | Promoting the release of glucagon-like peptide-1 (GLP-1), glucose-dependent insulin-releasing peptide (GIP) |
| *Radix Puerariae* ^[47]^ | Puerarin | Reducing fat differentiation, alleviating insulin resistance, lowers blood levels of glucose, pressure, and lipids and enhances antioxidation | Inhibiting expression of adipogenic differentiation protein (ADRP). Protecting islet β cells, improving liver function, and activating the Akt pathway downstream of insulin receptors. Inhibit the expression of hepatic acetyl coenzyme A carboxylase (ACC) mRNA, alleviate IR by regulating the hepatic ACC signaling pathway, and further promote glucose and lipid metabolism |
| *Herba Dendrobii* ^[48]^ | Dendrobium nobile Lindl.alkaloids | Protecting the islets | Decreasing the phosphorylation levels of JNK Thr183 / Tyr185, and increasing the phosphorylation levels AKT ser473 in islet |
| *Radix Astragali seu Hedysari* ^[49-51]^ | Astragalus polysaccharide | Lowers blood glucose, lowers lipids, has an anti-inflammatory effect, and improves islet function, anti-oxidation, protection of renal tissue, protecting cardiomyocytes | Increasing the activity of SOD, inhibiting the level of 8-OHdG, MDA, and ROS, inhibiting the PKC, MAPK and JAK-STAT pathway; decreasing the expression of CTGF, ET-1, TGF-² ; decreasing the expression of NF-º B, MCP-1, and angiopoietin receptor Tie-2; up-regulation of MMP-9, increasing the activity of SOD, GSH, and inhibiting the level of MDA, activating Na+-K+-ATP enzymes, improve renal injury by interfering with signal transduction and inflammatory reactions, Astragaloside IV can increase the activity of fork-head box transcription factor 1 by inhibiting the phosphatidylinositol 3-kinase (PI3K)/protein kinase B signaling pathway. |
| *Radix Notoginseng* ^[52-54]^ | Panax notoginseng saponins | Protecting the retina, anti-oxidation of retina, inhibition of renal podocyte injury | Decreasing the expression of glial fibrillary acidic protein in the retina; inhibiting expression of Nogo receptors in the retina RGC; up-regulation of nephrin, ±3-integrin, and ² 1integrin |
| *Ginseng Radix et Rhizoma* ^[55-58]^ | Panax ginseng C. A. Mey | Enhancing learning and memory, strengthening the heart, anti-shock properties, enhancing immune function, and delaying aging. | Ginsenoside Re can selectively promote enzyme activity in the islet β cell plexus and acetylcholinesterase (AchE) activity in the hippocampus. Ginsenoside ­Rh2 can stimulate nerve endings to release acetylcholine, activating islet cell M receptors and promoting insulin secretion. Ginsenoside ­Rb1 also enhances the activity of islet β cells. Ginseng Radix et Rhizoma improve hepatocyte glycogen synthesis, promote glucose utilization, and reduce gluconeogenesis, thereby lowering blood glucose. Binding with a β-adrenoceptor to trigger the cyclic adenosine monophosphate signaling pathway and then promote aerobic glycolysis of mitochondrial glucose. |
| *Dioscoreae Rhizoma* ^[59-65]^ | Dioscorea opposita Thunb | Hypoglycemic, hypolipidemic, and antioxidant | Reduce blood glucose levels induced by alloxan and exhibits a specific concentration of dependence. Increasing insulin secretion and improving the function of damaged islet β cells, increase C-peptide values, regulate cholesterol levels, triacylglycerol, and other related blood lipid indicators, reduce blood glucose levels. |
| *Mori Folium* ^[56, 66]^ | Morus alba L. | Lowering blood glucose, regulating blood lipids, clearing away oxygen free radicals, and anti-virus activities | Reduces the levels of serum free fatty acid (FFA), inhibits apoptosis induced by FFA, improves energy homeostasis based on the expression of adenylate kinase 2 (AK2) protein and peroxidase proliferator-activated receptorγcoactivator 1α (PGC1α) protein, and has an anti-diabetes effect. |
| *Codonopsis Radix* ^[67-71]^ | Codonopsis pilosula (Franch.) Nannf. | Regulates blood glucose, enhances immunity, has anti-hypoxia and anti-stress effects and delays aging | Scavenge free radicals in vivo and in vitro and reduce the damage caused by oxygen free radicals to islet β cells by enhancing antioxidant effects, protecting β cells, and attenuating IR. Increase the cell proliferation ability of the diabetes vascular endothelial cell injury model, thereby inhibiting diabetes vascular endothelial cell injury, and its joint silencing information regulatory protein 4 (SIRT4) can inhibit oxidative stress and mitochondrial apoptosis, thus inhibiting diabetes vascular endothelial cell apoptosis in vitro. |
| *Ophiopogonis Radix* ^[72]^ | Ophiopogon japonicas (L.f) Ker-Gawl. | Lower blood glucose levels, enhance immunity, delay skin aging, and provide anti-inflammation and anti-tumor effects | Promote the transport and utilization of glucose by adipocytes, reduce FBG levels and inhibit IR. It enhances the sensitivity of exogenous insulin and increases the content of hepatic glycogen and skeletal muscle glycogen in blood. |
| *Acupuncture* ^[73-75]^ | A stimulation therapy applied to specific acupoints (such as Sanyinjiao, Zusanli, Pishu, Yinlingquan, etc.) to reach therapeutic goals | Lower blood glucose, reducing the level of inflammatory factors, and attenuate IR | It affects the bioavailability of substances taken internally and regulates the level of plasma free fatty acids (FFAs), impacting insulin sensitivity. Promote the aggregation of leptin in the hypothalamus, increase energy consumption, reduce the levels of serum glycerol and cholesterol. |
| *Acupoint sticking* ^[76-78]^ | Chinese herbs are made into medicinal cakes or pastes, which are directly applied to the corresponding acupoint points (such as *Shenque, Yongquan*) on the body | Improve the diabetes symptoms (such as constipation and abdominal distension) | The therapeutic agent acts on the body by absorption through the skin and the mucous membrane, stimulating the Qi and blood of meridians and collaterals, adjusting the Zang-Fu, balancing Yin and Yang, and reconciling Qi and blood. This helps to adjust the gastrointestinal Qi mechanism, improving the stool form, increasing gastrointestinal motility, alleviating abdominal distension, promoting defecation, exhaustion, and gastrointestinal emptying. Avoid the first pass effect and the stimulation of the drugs on the gastro-intestinal tract, the aim of treating diseases is achieved through transdermal absorption. |
| *TCM foot bath combined with acupoint therapy* ^[79-81]^ | TCM formulae are decocted for use in a foot bath. Acupuncture points, meridians, and local foot reflex zones are stimulated through methods such as massage, acupuncture, and applying poultices. Care is taken to control the water temperature to maintain it around 38℃. | Dilating blood vessels, increasing blood flow speed, and promoting microcirculation | Promote microcirculation, improve skin permeability, and increase drug absorption. Improve microcirculation, relieve inflammation, and reduce the production of free radicals and maintain the dynamic balance of it. |
| *Herbal fumigation* ^[82]^ | The affected limb is soaked in warm medicinal solution, allowing the medicine to directly act on the skin. Care is taken to control the water temperature to maintain it around 38℃. | Increasing blood supply to the surrounding tissues, repairing peripheral nerve damage, and improving blood circulation and nerve function | The effective ingredients penetrate the skin directly to the diseased area or enter the bloodstream, the medicinal components are more likely to act on the diseased site. |
| *Auricular therapy* ^[83, 84]^ | Wang Buliuxing seeds or magnetic beads were used and pressed to the corresponding auricular points | Improve blood glucose indicators, alleviate symptoms of insomnia | By stimulating the nerves to regulate the secretion of insulin, it promotes pancreatic alpha cells to secrete insulin in advance, thereby lowering blood sugar, eliminating and reducing clinical symptoms. Excite the nerve receptors and sensory nerve endings, regulate the cerebral cortex, and restore the physiological sleep rhythm. |
| *Acupoint catgut embedding* ^[85, 86]^ | The catgut, collagen thread, or polymer line is buried in the target acupoints, and the continuous stimulation of the catgut itself and the acupoint is used to prevent and treat diseases. | Lasting stimulation of the acupoints, increase insulin sensitivity to make patients feel full to reduce food intake | Increasing the expression of peroxisome proliferators activated receptorγ PPAR-γ mRNA in adipose tissue and inhibiting the expression of visfatin in visceral adipose tissue so as to achieve multiple biological functions such as producing insulin, regulating immune and inflammatory responses, and promoting adipogenic differentiation and synthesis.It also can reduce uric acid levels in the blood of the obese patients and increase insulin sensitivity. |
| *Natural components from TCM herbs* ^[87]^ | Flavonoids, terpenoids, alkaloids, and other compounds | Reduce blood glucose, antioxidation, anti-inflammation, regulation of glucose and lipid metabolism, and restoration of pancreatic islet cell function | Enhancing the activity ofantioxidant enzymes, inhibiting the production of ROS and reducing the content of MDA, scavenging free radicals in the human body. These natural components show potency to regulate glucose and lipid metabolism by activating AMPK, inhibiting α-Amy and α-Glu activity, and regulating the PPAR signaling pathway. Additionally, these natural compounds can promote the recovery of pancreatic islet cell function by inhibiting inflammatory signaling pathway to protect β-cell function, regulating AMPK activity and related gene expression to enhance peripheral insulin sensitivity and improve IR, and regulating the PI3K/Akt signaling pathway to protect islet cells. Flavonoids can reduce blood glucose mainly by regulating AMPK activity, regulating PPARγ, and inhibiting α-Glu activity. |

REFERENCES

[1]Tian J, Jin D, Bao Q, et al. Evidence and potential mechanisms of traditional Chinese medicine for the treatment of type 2 diabetes: A systematic review and meta-analysis. *Diabetes Obes Metab*. 2019;21(8):1801-1816.

[2]Li Y, Fan X, Wang Y, et al. Therapeutic effect and mechanism of Gegenqianlian decoction on type 2 diabetic rats. *Acta Pharmaceutica Sinica.* 2013;48:1415-1421.

[3]Li J, Gao T, Song Q, et al. Study on Mechanism of effect of Gegenqinlian decoction on lowering blood sugar and lipid of type 2 diabetic rats. *Journal of Hubei University of Chinese Medicine.* 2015;17:7-9.

[4]Zhang C, Ma G, Deng Y, et al. Effect of Gegen Qinlian decoction on LPS, TNF-±,IL-6,and intestinal flora in diabetic KK-Ay mice. *Chinese Traditional and Herbal Drugs.* 2017;48:1611-1616.

[5]Meng X, Liu X, Tan J, et al. From Xiaoke to diabetes mellitus: a review of the research progress in traditional Chinese medicine for diabetes mellitus treatment. *Chin Med*. 2023;18(1):75.

[6]Guo Y, Du J, Jiang M. Effect of Baihutang regulating IRS-1/PI3K/Akt signal pathway on blood glucose, blood lipid metabolism and vascular remodeling in type 2 diabetic rats. *Chin J Exp Tradit Med Formulae*. 2021;27(1):23-30.

[7]Weng L, Chen L, Xu Y, et al. Chemical compositionand pharmacological action of Rhizoma Anemarrhenae. *Jilin J Chin Med*. 2018;38(1):90-92.

[8]Feng F. A review on pharmacological effects of Rhizoma Anemarrhenae. *Clin J Chin Med*. 2017;9(12):133-137.

[9]Meng X, Ma J, Kang AN, et al. A novel approach based on metabolomics coupled with intestinal flora analysis and network pharmacology to explain the mechanisms of action of Bekhogainsam decoction in the improvement of symptoms of streptozotocin-induced diabetic nephropathy in mice. *Front Pharmacol*. 2020;11:633.

[10]Peng L, Diao J, Wang L. Research progress on pharmacological effect of Banxia Xiexin decoction. *China Med Her*. 2019;16(36):37-39.

[11]Lan X. Effect of Banxia Xiexin decoction combined with metformin in the treatment of type 2 diabetes patients with insomnia. *Diabetes New World*. 2021;24(23):63-66.

[12]Yang J. Banxia Xiexin decoction on insulin resistance and expression of IL-6, TNF-a, FFA and NO in type 2 diabetic rats. *Chengdu University of Traditional Chinese Medicine*, 2017.

[13]Cui W. Influence of Banxia Xiexin decoction on insulin resistance of diabetic rats. *Acta Chin Med*. 2016;31(7):1008-1011.

[14]Bao Y, Xie W, Wang J. Research progress of Erchentang. *Chin J Exp Tradit Med Formulae*. 2019;25(23):9-18.

[15]Wu T, Gao B, Lin S, et al. Impacts of different herbal formulas on blood lipid in insulin resistance model rats. *World J Integr Tradit West Med*. 2009;4(7):470-472.

[16]Li X, Yang S, Nie M, et al. Effect of pingtang recipe on blood glucose, lipid, resistance of insulin, liver function and changes of hepatic lipid in rats with type 2 diabetes mellitus complicated with fatty liver. *Chin J Tissue Eng Res*. 2006;11:77-80.

[17]Zhao T, Zhan L. Research progress on the mechanism of Erchen decoction in metabolic diseases. *Mod Tradit Chin Med Materia Medica-World Sci Technol*. 2021;23(4):998-1005.

[18]Liu L, Zhang Y, Zhu Z, et al. Yuquan pill enhance the effect of Western medicine in treatment diabetic nephropathy: a protocol for systematic review and meta-analysis. *Medicine (Baltimore)*. 2021;100(42):e27555.

[19]Mai X, Deng X, Luo X, et al. Research progress of Yuquan pill in the treatment of type 2 diabetes. *China’s Naturopathy*. 2020;28(18):107-109.

[20]Peng S, Xie Z, Zhang X, et al. Efficacy and safety of the Chinese patent medicine Yuquan pill on type 2 diabetes mellitus patients: a systematic review and meta-analysis. *Evid Based Complement Alternat Med*. 2021;2021:2562590.

[21]Xue B, Wang X, Liu H, et al. Research progress of Danggui Liuhuang decoction. *Guiding J Tradit Chin Med Pharm*. 2020;26(13):162-167.

[22]Gao Z, Li Q, Wu X, et al. New insights into the mechanisms of Chinese herbal products on diabetes: a focus on the “bacteria-mucosal immunity-inflammation-diabetes” axis. *J Immunol Res*. 2017;2017:1813086.

[23]Li H, Meng X. Research progress on chemical constituents and pharmacological activities of Rehmannia glutinosa. *Drug Eval Res*. 2015;38(2):218-228.

[24]Cao S, Li Y, Ding H. Research progress on Yunyu decoction in the treatment of type 2 diabetes mellitus. *Chin Med Mod Distance Educ China*. 2017;15(12):156-158.

[25]Xue G, Jin M, Li S, et al. Chemical constituents from Rehmannia Radix Praeparata and their biological activities in vitro. *Chin Tradit Pat Med*. 2018;40(12):2689-2692.

[26]Yang L, Jiang H, Yang B, et al. Isolation and identification of chemical constituents from the root of Achyranthes bidentata BL. *Inform Tradit Chin Med*. 2012;29(1):22-24.

[27]Wang T, Wang Y, Sun A, et al. HPLC for simultaneous determination of various quota components in Yunvjian. *J Tianjin Univ Tradit Chin Med*. 2017;36(3):214-218.

[28]Yao F, Sun L. Advance on pharmacological activities and structural modifications of mangiferin. *Chin J Exp Tradit Med Formulae*. 2014;20(12):248-52.

[29]Zhang L, Sun D, Tu W, et al. Study on determination of β-ecdysterone and fingerprints of Achyranthes bidentata Bl. from different areas. *Nat Prod Res Dev*. 2013;25(4):500-505.

[30]Nagaoka T, Shirakawa T, Balon TW, et al. Cyclic nucleotide phosphodiesterase 3 expression in vivo: evidence for tissue-specific expression of phosphodiesterase 3A or 3B mRNA and activity in the aorta and adipose tissue of atherosclerosis-prone insulin-resistant rats. *Diabetes*. 1998;47(7):1135-1144.

[31]Dai B, Wu Q, Xiao Z, et al. Effects of Liuwei Dihuang decoction and its water-extracted alcohol-soluble parts on PI3K/Akt signaling pathway in adipose tissue of type 2 diabetes model rats. *Chin Tradit Pat Med*. 2016;38(2):428-430.

[32]Wang Y. Effect of Liuwei Dihuang decoction on T2DM and its effect on islet β cell. *China Continuing Med Educ*. 2020;12(33):164-167.

[33]Liu A, Zhang G, Huang J, et al. The effects of Jinlida Granules on the muscle oxidative stress and the expression of SIRT3 in diabetic rats. *Journal of Clinical and Experimental Medicine.* 2017;16:323-325.

[34]Liu Y, Zang S, Song G, et al. Effects of Chinese medicine Jinlida on hepatic oxidative stress and JNK, p38MAPK signaling pathway in rats fed with high-fat diet. *China Journal of Traditional Chinese Medicine and Pharmacy.* 2015;30:2156-2159.

[35]Zang S, Liu Y, Song G, et al. Effects of Jinlida Granules on lipid accumulation in skeletal muscle and mito-chondrial function in insulin resistant rats. *Chinese Trad Patent Med*. 2014;36:1371-1376.

[36]Jin X, Zhang H, Cui W, et al. Effect of Jinlida Granules on SREBP-1c in skeletal muscle in fat-induced insulin resistance mice. *Chinese Trad Patent Med.* 2015;37:705-710.

[37]Wang C, Dai X, Zhang D, et al. Jinlida granules improve dysfunction of hypothalamic-pituitary-thyroid axis in diabetic rats induced by STZ. *Biomed Res Int.* 2018;2018:4764030.

[38]Wang D, Tian M, Qi Y, et al. Jinlida granule inhibits palmitic acid induced-intracellular lipid accumulation and enhances autophagy in NIT-1 pancreatic beta cells through AMPK activation. *J Ethnopharmacol.* 2015;161:99-107.

[39]Zhou J, Wu Y, Liu S, et al. Effects of main chemical components of Xiaoke Pills on glucose and lipid metabolism of insulin resistant adipocytes. *J Chinese Medicinal Materials*. 2015;38:1270-1276.

[40]Qian J, Li M, Wu J, et al. The inhibiting effect of compositions from Xiaoke Pill on the ±-glucosidas. *Chinese J Exp Trad Medical Formulae.* 2012;18:173-176.

[41]Geng C, Liu J, Zhou Q, et al. Impacts on blood lipid, insulin and pancreas treated with the ingredients of Xiaoke pill in GK rat. *World J Integrated Trad Western Med.* 2014;9:822-825.

[42]Zhou J, Xun Y, Huang S, et al. Effect of Compound Danshen dropping pill on Islet ² Cell function in type 2 diabetic rat. *Chinese Trad Patent Med.* 2015;37:1807-1810.

[43]Chen P, Zheng Q, Chen W, et al. The effects of Danshen dripping pills on the expressions of HIF-1± and VEGF in renal tissues of type 2 diabetic rat. *Chinese J Health Care Med*. 2011;13:200-203.

[44]Lu W, Zhang X, Cheng S, et al. Protective effect of compound Danshen dropping pill on early diabetic nephropathy in rats and its mechanism. *J Xi’an Jiaotong University (Medical Sciences)*. 2016;37:128-133.

[45]Song B, Liu X. Effect of emodin on expression of PPAR-± and PPAR-³ in adipose tissue of KKAy diabetic mice. *J Beijing University Trad Chinese Med.* 2012;35:692-695.

[46]Cai C, Wang H, Wang S, et al. Therapeutic effect of Rehmannia glutinosa polysaccharide on obese diabetic rats and its effect on serum GLP-1 and GIP levels. *Chinese J Gerontol.* 2013;33:4506-4507.

[47]Sun W, Zheng X, Xu Q, et al. Effects of puerarin on ADRP gene expression in adipose tissue of type 2 diabetic rat. *China J Chinese Materia Medica.* 2008;16:2026-2028.

[48]Chang H. Effects of Dendrobium candidum on the phosphorylation of JNK and AKT proteins in pancreatic islet tissue of type 2 diabetic rats. *Chinese Pharmaceutical Affairs.* 2015;29:54-57.

[49]Fan Y, Li N, Sun Y, et al. Effect of effective fraction of astragalus membranaceus on Na-K-ATPase activity and AMPK protein expression in diabetic rats. *China J Trad Chinese Med Pharm.* 2012;27:2660-2663.

[50]Sun J, Fan Y, Li N, et al. Effect of effective fraction of astragalus membranaceus on interleukin-1² and interleukin - 4 in diabetic rats. *China J Trad Chinese Med Pharm.* 2012;27:1908-1910.

[51]Lv Z, Liu L, Wu Z, et al. Inhibitory effect of different doses of Astragalus membranaceus on oxidative stress in diabetic rats. *Shanghai J Trad Chinese Med.* 2015;49:68-71.

[52]Zhu D, Liu X. Protective effect of Panax notoginseng saponins on retinal ganglion cells in diabetic rats. Tianjin Med J 2013;41:1103-1105.

[53]Zhou J, Ai Z, Sun W, et al. Study on the protective effect of Panax notoginseng saponins on podocyte protection in diabetic nephropathy mice. *China J Trad Chinese Med Pharm.* 2014;29:1316-1321.

[54]Zhao L, He T, Peng J, et al. Protective effect of Panax notoginseng on diabetic retinopathy in diabetic rats. *Chinese J Diabetes.* 2014;22:656-659.

[55]Wang C, Tao Q, Lou X. Progress in research on the mechanism of Chinese medicine in the treatment of diabetes. *Glob Tradit Chin Med*. 2022;15(1):152-158.

[56]Han X. Exprimental progress of research of mechanism of action of single chinese herb and extract for type II diabetes. *Contemp Med Symp*. 2019;17(8):9-11.

[57]Sun L, Liang X. Progress of experimental research on hypoglycemic mechanism of traditional Chinese medicine. *China J Tradit Chin Med Pharm*. 2007;11:789-791.

[58]Gu H, Wu J, Bai S, et al. Influence of ginseng two-ingredient capsule on blood glucose and blood lipids in diabetic rats. *Chin J Exp Tradit Med Formulae*. 2011;17(7):132-135.

[59]Chen M, Liu W, Chou G, et al. Research progress on chemical constituents and pharmacological activities of Dioscorea opposita thunb. *Acta Chin Med Pharmacol*. 2020;48(2):62-66.

[60]Jia K, Gao Y, Chen X, et al. Application progress of metabonomics in the treatment of type 2 diabetes mellitus. *China Med Her*. 2019;16(24):36-39.

[61]Lv J, Wei P, Bai F. Effects of Dioscoreae Rhizoma polysaccharide on platelet and enzyme activities in type 2 diabetic rats. *Chin J Gerontol*. 2017;37(13):3186-3187.

[62]Zhi F, Xing Q, Wang Y, et al. Effect of Dioscorea opposita Thunb. Polysaccharide on glycolipid metabolism and oxidative stress in type 2 diabetic rats. *Food Sci*. 2017;38(5):262-266.

[63]Xing W, Hou J, Han H, et al. Effects of Yam polysaccharide on blood glucose and serum antioxidant capacity in type i diabetic mice. *Food Res Dev*. 2014;35(17):107-110.

[64]Hu G, Yang B, Zhang Z. Effect of polysaccharides of rhizoma on blood glucose and insular function in diabetic rats. *Shandong J Tradit Chin Med*. 2004;4:230-231.

[65]Li X, Pei L, Chen Y, et al. The influence of Dioscorea Batatas polysaccharide on the glycolipid metabolism and oxidative stress of diabetic rats induced by STZ. *Chin J Gerontol*. 2014;34(2):420-422.

[66]Huang L, Liu T, Sun W, et al. Effect and mechanism of flavonoids from Mori folium on blood glucose level in diabetic rats. *Chin J Exp Tradit Med Formulae*. 2018;24(16):152-156.

[67]Huang Y, Zhang Y, Kang L, et al. Research progress on chemical constituents and their pharmacological activities of plant from Codonopsis. *Chin Tradit Her Drugs*. 2018;49(1):239-250.

[68]Xu A, Zhang Z, Ge B, et al. Study effect and its mechanism on resisting senility of PCP. *Chin J Mod Appl Pharm*. 2006;S2:729-731.

[69]Li G, Yang S. Extraction of Codonopsis Pilosula polysaccharide and its effects of antiactive oxygen free radicals. *Chem World*. 2001;42(8):421-422+434.

[70]He K, Li X, Chen X, et al. Evaluation of antidiabetic potential of selected traditional Chinese medicines in STZ-induced diabetic mice. *J Ethnopharmacol*. 2011;137(3):1135-1142.

[71]Su H, He J, Bao L, et al. Effects of radix codonopsis polysaccharides combined with sirtuin 4 on apoptosis of vascular endothelial cells induce by high glucose in vitro. *Chin J Microcirc*. 2020;30(3):11-18.

[72]Li J, Su W, Wang Y, et al. Protective effects of Ophiopogonis japonicas extract on experimental type 2 diabetic rats. *Acta Sci Natur Univ Sunyatseni*. 2017;56(3):119-124.

[73]Song S, Li R, Cao B, et al. Effect of electro-acupuncture on levels of blood glucose, lipids and leptin in rats with type 2 DM. *J Clin Acupunct Moxibustion*. 2020;36(4):87-90.

[74]Cai YJ, Liu X, Li GZ. Research progress on the application of Taijiquan in type 2 diabetes mellitus. *China J Tradit Chin Med Pharm*. 2023;38(1):298-300.

[75]Firouzjaei A, Li GC, Wang N, et al. Comparative evaluation of the therapeutic effect of metformin monotherapy with metformin and acupuncture combined therapy on weight loss and insulin sensitivity in diabetic patients. *Nutr Diabetes*. 2016;6(5):e209.

[76]Yan L, Liu H, Yan R, et al. Effect of traditional Chinese medicine external therapy for functional constipation: a meta-analysis. *Am J Transl Res*. 2023;15(1):13-26.

[77]Ding M, Huang HR, He LH. Meta-analysis on acupoint patching for the treatment of diabetic constipation. China Medicine and Pharmacy. 2023;13(15):94-97+140.

[78]Chen YX, Yun J, Gong TL, et al. Meta-analysis of function on acupoint application in patients with diabetic constipation. *Chinese Journal of Ethnomedicine and Ethnopharmacy*. 2022;31(23):98-103.

[79]Fu Q, Yang H, Zhang L, et al. Traditional Chinese medicine foot bath combined with acupoint massage for the treatment of diabetic peripheral neuropathy: A systematic review and meta-analysis of 31 RCTs. *Diabetes Metab Res Rev*. 2020;36(2):e3218.

[80]Gu C, Wang LY. Meta analysis of the effect of traditional Chinese medicine foot bath combined with acupoint therapy on diabetic peripheral neuropathy. *Chinese Journal of Modern Drug Application*. 2022;16(17):166-170.

[81]Wang AN, Gao H, Yang YF, et al. A systematic review of foot bath of traditional Chinese medicine combining mecobalamin on diabetic peripheral neuropathy. *Journal of Liaoning University of Traditional Chinese Medicine*. 2019;21(2):98-103.

[82]Chen S, Song RP, Su Y, et al. A meta-analysis of TCM medicine fumigation and washing in the adjuvant treatment of diabetic painful neuropathy. *Clinical Journal of Chinese Medicine*. 2023;15(6):23-27.

[83]Yang Q, Jing L, Wang ZW, et al. Meta-analysis ofauricular plaster therapy combined with traditional Chinese medicine for pre-diabetes. *Journal of Hunan Normal University (Medical Edition)*. 2022;19(6):113-118.

[84]Zhu WL, Shi H, Li J, et al. Meta-analysis of the effect of auricular point plaster therapy on insomnia in patients with diabetes. *Journal of Baotou Medical College*. 2018;34(8):90-94.

[85]Wujie YE, Jingyu X, Zekai YU, et al. Systematic review and meta-analysis of acupuncture and acupoint catgut embedding for the treatment of abdominal obesity. *J Tradit Chin Med*. 2022;42(6):848-857.

[86]Jiali W, Lily L, Zhechao L, et al. Acupoint catgut embedding versus acupuncture for simple obesity: a systematic review and Meta-analysis of randomized controlled trials. *J Tradit Chin Med*. 2022;42(6):839-847.

[87]Li X, Geng-Ji JJ, Quan YY, et al. Role of potential bioactive metabolites from traditional Chinese medicine for type 2 diabetes mellitus: An overview. *Front Pharmacol*. 2022;13:1023713.
